# Development of a novel cultivation technique for uncultured soil bacteria

**DOI:** 10.1038/s41598-019-43182-x

**Published:** 2019-04-30

**Authors:** Dhiraj Kumar Chaudhary, Altankhuu Khulan, Jaisoo Kim

**Affiliations:** Ecology Laboratory, Department of Life Science, Kyonggi University, Suwon, South Korea

**Keywords:** Microbiology techniques, Biodiversity

## Abstract

In this study, a new diffusion bioreactor was developed to cultivate hidden bacterial communities in their natural environment. The newly developed method was investigated to cultivate microbial communities from the forest soil, and the results were evaluated against traditional culture methods and compared to the results of a pyrosequencing-based molecular survey. The molecular analysis revealed that a diverse bacterial population was present in the soil sample. However, both the newly developed method and the traditional method recovered more than 400 isolates, which belonged to only four phyla: *Proteobacteria*, *Firmicutes*, *Actinobacteria*, and *Bacteroidetes*. Although these isolates were distributed over only four major phyla, the use of the newly developed technique resulted in the successful cultivation of 35 previously uncultured strains, whereas no such strains were successfully cultivated by the traditional method. Furthermore, the study also found that the recovery of uncultured bacteria and novel isolates was related to sampling season, incubation period, and cultivation media. The use of soil collected in summer, a prolonged incubation period, and low-substrate modified media increased the recovery of uncultured and novel isolates. Overall, the results indicate that the newly designed diffusion bioreactor can mimic the natural environment, which permits the cultivation of previously uncultured bacteria.

## Introduction

Studies of microbial 16S rRNA gene sequences have revealed that 4 × 10^6^ different microbial taxa are present per ton of soil and that 10^9^ cells are present per gram of soil^1–4^. With the development of next-generation sequencing techniques, along with extensive implementation of metagenomic tools, scientists are discovering novel taxa and evaluating the diverse microbial flora present in soil. However, overwhelming numbers of these microbial communities are not-yet-cultured on synthetic media *in vitro* and remain unexplored^5–7^.

These “unculturable” microorganisms represent a large untouched pool of species with novel biological and chemical properties^8^. Unculturable bacteria are metabolically active in their native environment but are unable to proliferate in laboratory media. The misnomer of “unculturable” does not suggest that these organisms can never be cultured, but rather, it indicates a lack of appropriate knowledge on their habitats, abiotic-biotic interactions, and ecological role in soil^6^. Currently, many scientists are investigating unculturable bacteria and have attempted to develop strategies to cultivate them. However, more than 99% of soil bacteria are still unculturable^9,10^. With the help of molecular techniques, the existence of the functional diversity of these uncultured microbes has consistently been demonstrated. The hidden potential of uncultured microbes to produce secondary metabolites and their biotechnological applications should be explored^11^. To achieve these goals, previously uncultured bacteria need to be cultivated in the laboratory. Unfortunately, this field of study is still in its infancy.

The major obstacle to bacterial growth in laboratory media is the failure to maintain a natural growth environment, and a lack of information regarding factors such as multiplication period, appropriate temperature, and nutrient conditions for growth makes some bacteria unculturable. The traditional approaches used to cultivate bacteria are focused on the use of nutrient-rich media and on creating an environment that benefits fast-growing species^6,9,10^. However, nutrient-rich media may be toxic to those microbes that survive under nutrient-poor conditions. The use of conventional techniques has been shown to favour fast-growing bacteria and undervalued slow-growing bacteria^5^. Additionally, most culture media use agar as a solidifying agent. In some cases, agar can inhibit the replication of microorganisms^12^. However, when used in place of agar, gellan gum has been shown to increase the growth rate of a number of microbes^13^. In addition, some organisms may require specific indicator agents (for example, a signal) to reveal the presence of an appropriate environment to initiate growth^6^. Some bacteria can survive only in the presence of helper organisms and helper agents. Helper agents such as siderophores solubilize iron, making it available to microorganisms that cannot proliferate without this activity. Similarly, some helper organisms may protect other organisms from toxic effects of the environment by removing oxidative stress^6^. These helper-dependent bacteria are unable to perform essential metabolic activities without helper agents and helper organisms in the growth environment^14^. In nature, the interaction between microbial communities and their metabolism is essential for their proliferation and depends on numerous physiological parameters, such as nutrients, pH, osmotic conditions, temperature, and several other factors^13^. Thus, it is impractical to mimic the natural conditions to cultivate bacteria without resolving the above-mentioned difficulties.

Metagenomics investigations have identified as-yet-uncultured bacterial phylogenetic clades, and in the past few years, a number of studies have attempted to cultivate these missing microbial candidates^7^. Some effective strategies have been developed to cultivate unculturable bacteria, including modifying nutrients and growth conditions, prolonging the incubation period, co-culturing with helpers, and simulating natural environments. In recent years, several advances have been made in cultivation techniques utilizing Transwell plates, optical tweezers and laser microdissection, microbioreactors, and diffusion chambers^5^. However, the existing techniques are still not sufficient to mitigate the known difficulties of cultivating hidden bacteria from soil. Therefore, the development of a wide variety of cultivation technologies that use novel approaches to mimic natural habitats is essential to allow for the replication of previously uncultured bacteria. In this study, a novel cultivation technique for uncultured soil bacteria was developed that allows for the growth of soil bacteria in their natural habitat. The newly developed technique utilizes a diffusion bioreactor that is incubated in a chamber to simulate the natural setting of the previously uncultured bacteria. The findings of this study contribute to the cultivation methodologies used for unculturable soil bacteria, allowing them to be extracted from soil and conveniently cultured in the laboratory.

## Methods

### Soil sampling

During a two-year period, soil was sampled 6 times (during February 2015, May 2015, July 2015, January 2016, June 2016, and August 2016) from the same geographical location of a forest inside the campus perimeter of Kyonggi University, Suwon, South Korea (GPS coordinates: 37°18′1.0368″N 127°2′17.1204″E). The surface soil was collected from the uppermost layer (0–10 cm deep) in a sterile zippered bag and stored at room temperature. The collected soils were sieved through a 2-mm mesh sieve to exclude pebbles and debris. Subsequently, the sieved soil was immediately utilized for cultivation, the preparation of soil extract and to mimic natural environments.

### Formulation of culture media

For the cultivation of soil bacteria, R2A, TSA, LB, NB, 50% diluted R2A, R2A-SE (1:1, v/v), and SCA-SE (1:1, v/v) culture media were used. In addition, J26-SE (1:1, v/v) medium was formulated in the laboratory by adding trace elements (SL-10), selenite tungstate, and soil extract (SE) (Table S1). SE has been widely used to isolate soil bacteria^15^. To prepare SE, 1 kg of soil was suspended in 2 L of distilled water (d/w) and shaken overnight on a shaker at room temperature. The soil suspension was allowed to settle, and the fluid was centrifuged at 3400 rpm for 10 min. Subsequently, the supernatant was filtered through a sterilized 0.2-µm membrane filter (Whatman filter paper, No. 2; GE Healthcare UK Limited). To prevent fungal contamination, the culture medium was supplemented with cycloheximide (50 µg/mL) during the cultivation period.

### Conventional cultivation technique

**Figure 1:**
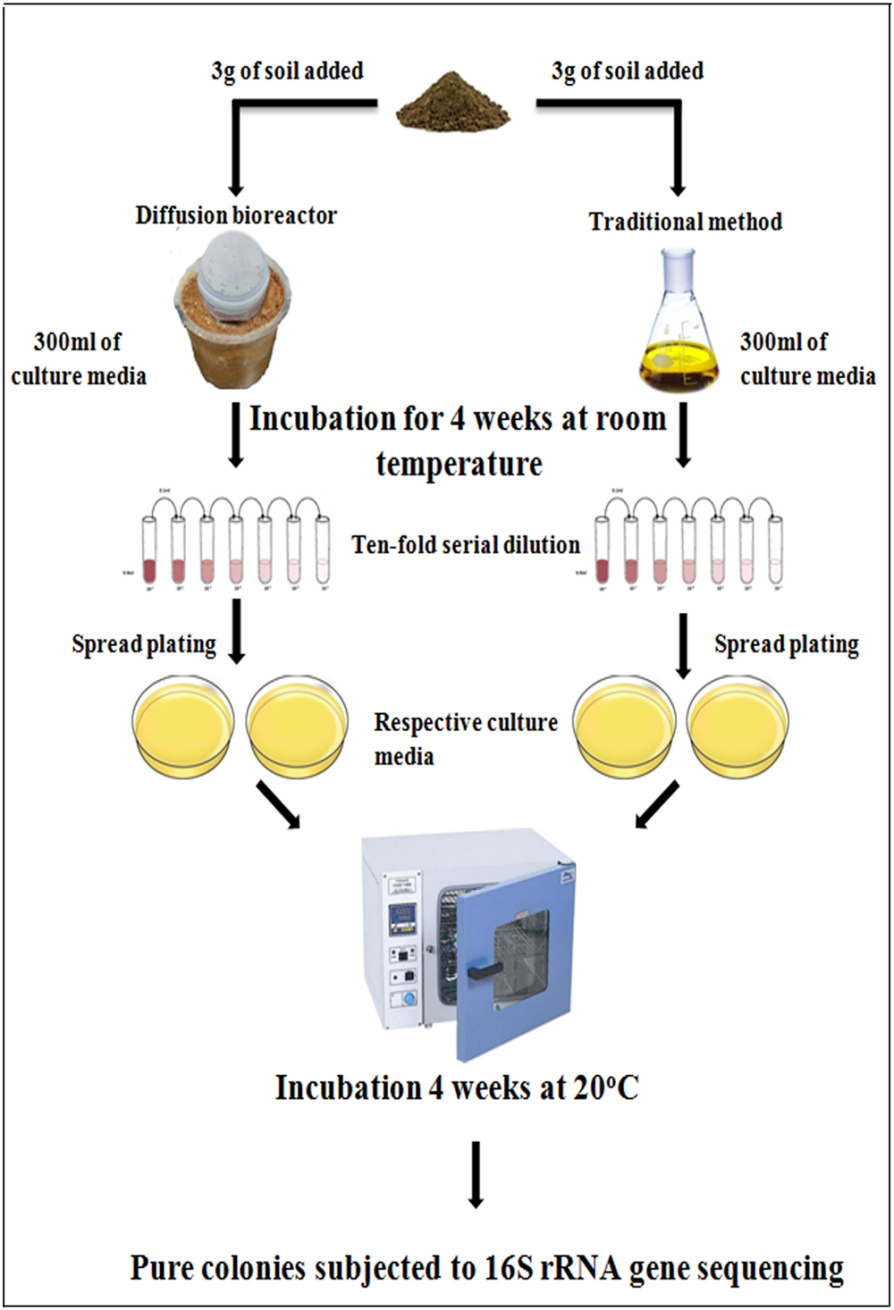
Scheme of the optimized protocol for the cultivation of previously uncultured microorganisms from forest soil.

For comparative analysis, soil bacteria were cultured using a conventional method. Three grams of soil was added to a conical flask containing 300 mL of medium, with duplicate flasks prepared for each medium (R2A, TSA, LB, NB, 50% R2A, R2A-SE, J26-SE, and SCA-SE). All of the flasks were incubated on a shaker at room temperature for 4 weeks (Fig. S1). Sampling was performed on weekly basis, and serial dilutions were generated with sterilized normal saline at 10^−1^ to 10^−6^ dilutions. A 100-µL aliquot of each serial dilution was spread onto agar medium plates and incubated aerobically at 25 °C for 4 weeks (Fig. 1). The resulting colonies were randomly picked and sub-cultured until pure colonies were obtained, which were subsequently identified through 16S rRNA gene sequencing as described below.

### Design of diffusion bioreactor and experimental setup for novel cultivation technique

**Figure 2:**
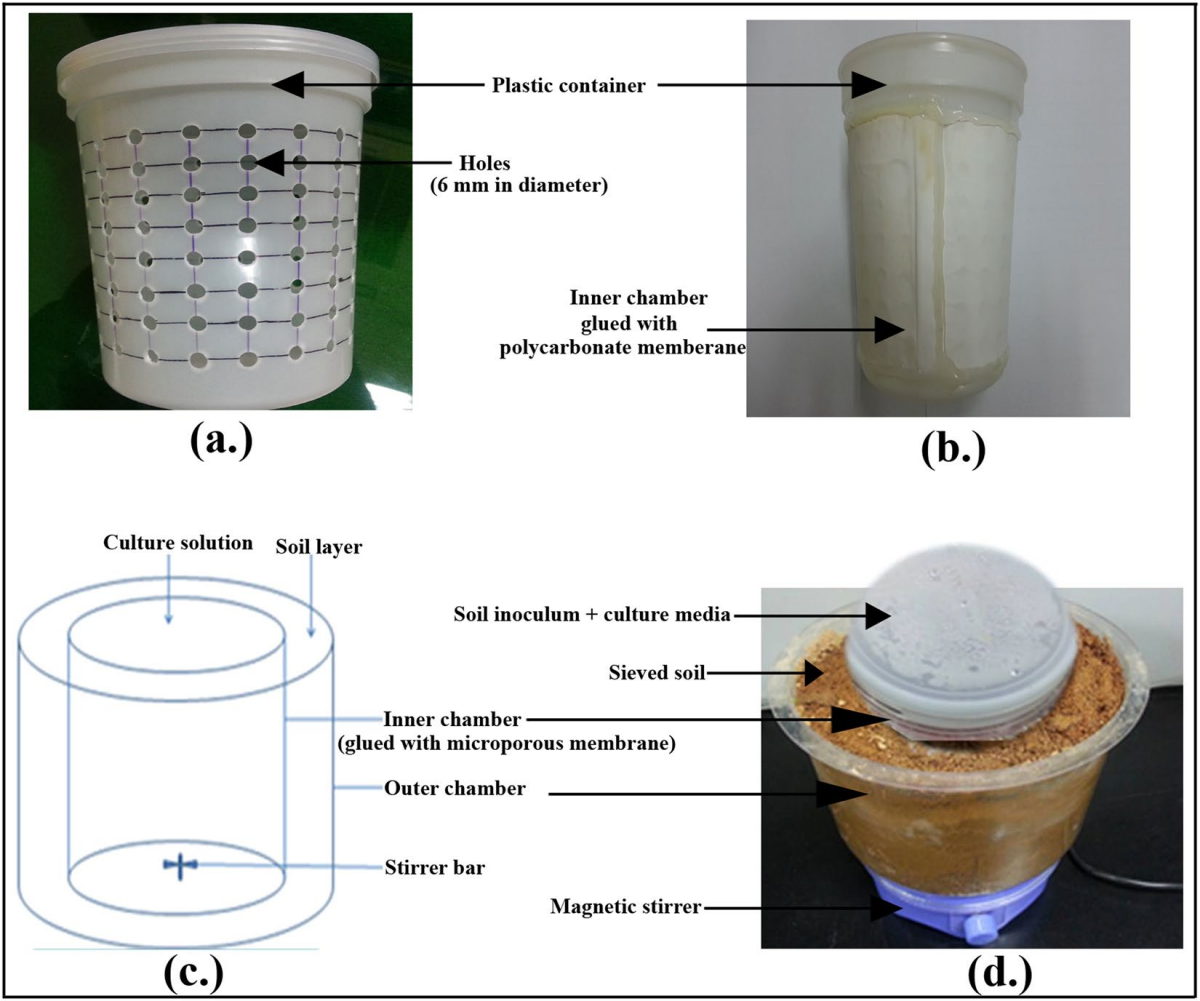
Design of the diffusion bioreactor for the cultivation of previously uncultured bacteria. (**a**) Plastic container perforated throughout with holes; (**b**) polycarbonate membrane glued inner chamber; (**c**) schematic diagram of the diffusion bioreactor; (**d**) overall experimental setup using the newly developed diffusion bioreactor.

In this study, the diffusion bioreactor comprised an inner chamber (2-L plastic container; 140-mm wide and 150-mm tall) and an outer chamber (4-L plastic container; 240-mm wide and 120-mm tall) (Fig. 2). A total of 160 holes (6 mm in diameter) were made in the wall of the inner chamber (Fig. 2a), and a polycarbonate membrane (0.4 µm pore size; GE Healthcare Life Science) was glued to the outer side of the inner chamber (Fig. 2b). All of the components used to build the diffusion bioreactor were sterilized in ethanol (70%, v/v), followed by drying under UV-light in a laminar flow hood for 24 h and subsequent rinsing in particle-free molecular grade water (Fisher Scientific, Hampton, NH). The inner chamber was placed inside the outer chamber, and the gap between the walls of the two chambers was filled with freshly sieved soil to allow for the natural soil environment to be maintained during the cultivation period. For cultivation, 3 g of soil and 300 mL of medium were added into the inner chamber (Fig. 2c). The diffusion bioreactors were setup in duplicate for each assayed culture medium (R2A, TSA, LB, NB, 50% R2A, R2A-SE, J26-SE, and SCA-SE), and the lid of the inner chamber was tightly closed and sealed with sealing tape (Fig. 2d). All of the bioreactors were stirred and incubated at room temperature for 4 weeks. During the cultivation period, the cells were provided access to their natural growth components, essential nutrients, and signalling compounds via diffusion. After incubating for 4 weeks, sampling and serial dilutions were performed as described above. One hundred-microliter aliquots of the serially diluted samples were plated onto agar plates for each medium and incubated aerobically at 25 °C for 4 weeks. Repeated subcultures were performed to obtain pure isolates, which were subsequently assayed by 16 S rRNA gene sequencing as described below (Fig. 1).

### PCR amplification, 16S rRNA gene sequencing, and phylogenetic analysis

Genomic DNA from pure isolates was extracted using Instagene Matrix (Bio-Rad, USA). PCR amplification of the 16S rRNA gene was conducted using the forward primer 27F (5′-AGAGTTTGATCMTGGCTCAG-3′) and the reverse primer 1492R (5′-TACGGYTACCTTGTTACGACTT-3′)^16^. PCR was performed in a 30-µL reaction mixture containing 20 ng of genomic DNA with the following cycling conditions: 95 °C for 2 min, followed by 35 cycles of 95 °C for 1 min, 55 °C for 1 min, and 72 °C for 1 min, with a final incubation at 72 °C for 10 min. The amplified PCR products were purified utilizing a multiscreen-filter purification kit (Millipore Corp., Bedford, MA, USA), and the purified products were sequenced using an Applied Biosystems 3730XL DNA analyser with a BigDye Terminator Cycle Sequencing Kit v.3.1 (Applied Biosystems). The primers 518F (5′-CCAGCAGCCGCGGTAATACG-3′) and 800R (5′-TACCAGGGTATCTAATCC-3′) were used for the sequencing reactions. The short sequence of the 16S rRNA gene was compiled using SeqMan (DNASTAR Inc.).

To determine the closest phylogenetic neighbours of the isolates, all of the sequenced 16S rRNA genes were compared using BLAST (https://blast.ncbi.nlm.nih.gov/Blast.cgi) and the EZBioCloud server (https://www.ezbiocloud.net)^17^. The following uniform cut-off values were implemented to infer novel taxa: species (99.0% ≥ x ≥ 96.5%), genus (96.5% > x ≥ 90%), family (90% > x ≥ 81.7%), order (81% > x ≥ 70%), and class (70% > x ≥ 65%), where ‘x’ represents the distance value^18,19^. Phylogenetic trees were constructed using MEGA6^20^ after generating a multiple alignment with the sequences of closely related members using CLUSTAL X 2.1^21^ and removing the gaps at the 5′ and 3′ ends using BioEdit^22^. The tree was inferred using the maximum-likelihood algorithm^23^, and the topology of the tree was calculated based on 1,000 resamplings^24^.

Statistical analysis was performed to calculate mean, standard deviation, and standard error. Significant differences (*p* < 0.05) were determined using two-way ANOVA. All of the statistical analyses were conducted using Microsoft Office Excel 2013 and OriginPro 8.5.

### DNA extraction from soil

Genomic DNA was extracted from soil samples (0.5 g) using a FastDNA SPIN kit (MP Biomedicals, France) following manufacturer’s protocol. The DNA was extracted in triplicate, and the quality of the extracted DNA was assessed through 1.5% agarose gel electrophoresis. Furthermore, the purity and concentration of the DNA samples were determined using a MaestroNano spectrophotometer (MaestroGen; Model name: MN-913).

### PCR amplification and pyrosequencing

The extracted soil DNA was used to PCR amplify the V1-V3 region of the bacterial 16S rRNA gene with the primers 27F and 518R. PCR amplification was conducted following previously described conditions^25^ and with the following thermocycling conditions: 95 °C for 5 min, followed by 30 cycles of 95 °C for 30 s, 55 °C for 30 s, and 72 °C for 30 s, with a final incubation at 72 °C for 5 min. The resulting amplicons were assessed by 1.5% agarose gel electrophoresis and observed under UV light. The PCR products were purified using a QIAQuick PCR Purification Kit (Qiagen, USA) and were subsequently used for pyrosequencing, which was performed by Chunlab, Inc. (Seoul, Korea), utilizing a Roche/454 GS FLX Titanium platform following the manufacturer’s instructions.

### Analysis of pyrosequencing data

The pyrosequencing data were analysed following previously described bioinformatics procedures^25–27^. All of the raw sequencing reads from the various soil samples were sorted and separated by the unique barcode sequences. Sequences with short lengths (<300 bp) and those with more than two ambiguous nucleotides were omitted prior to analysis^28^. The amplicons that were nonspecific and did not match the 16S rRNA gene database using the hidden Markov model and EZBioCloud server^17^ were excluded. Additionally, all of the sequence reads were screened for chimaeras using BLAST. All of the resulting sequence reads were assigned to an appropriate taxonomic position after comparisons with the EZBioCloud and GenBank databases (https://www.ncbi.nlm.nih.gov/genbank). The following cut-off values were used for the taxonomic allocation of the sequence reads: species (x ≤ 0.03), genus (0.03 < x ≤ 0.05), family (0.05 < x ≤ 0.1), order (0.1 < x ≤ 0.15), class (0.15 < x ≤ 0.2), and phylum (0.2 < x ≤ 0.25), where the ‘x’ represents distance value^2,29^.

## Results

### Overall bacterial diversity in the soil sample

**Figure 3:**
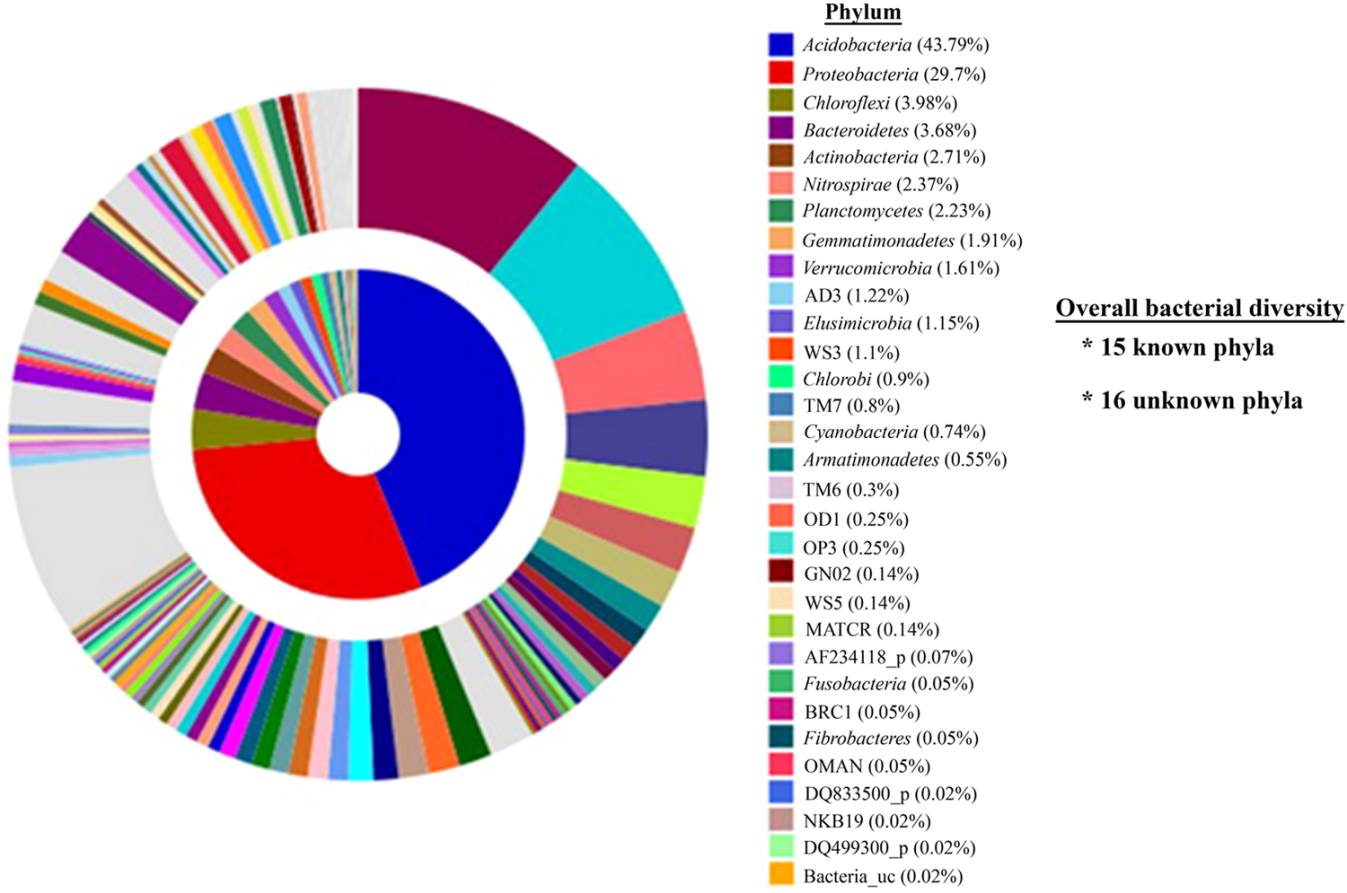
Relative abundances and taxonomic identification of bacteria revealed by pyrosequencing analysis. A total of 31 bacterial phyla were detected, 15 of which were previously known phyla and 16 were unclassified phyla.

**Figure 4:**
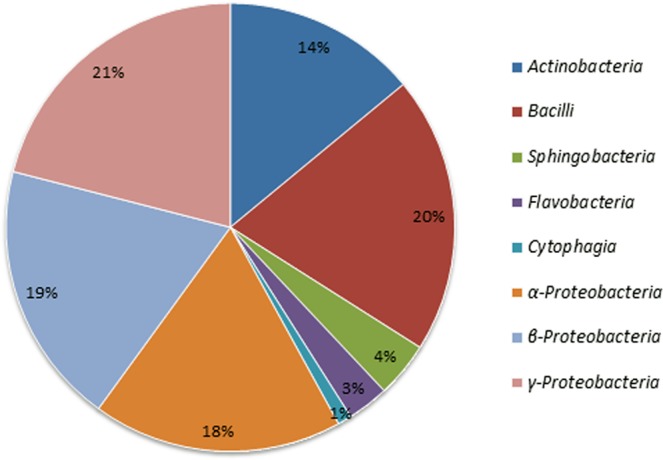
Taxonomic distribution of total pure cultured bacteria isolated from forest soil samples. The bacterial diversity of pure isolates was associated with one of eight classes: *Actinobacteria*, *Bacilli*, *Sphingobacteria*, *Flavobacteria*, *Cytophagia*, *α-Proteobacteria*, *β-Proteobacteria*, and *γ-Proteobacteria*.

The molecular analysis revealed a high bacterial diversity present in the forest soil. The 16S rRNA gene sequences obtained through pyrosequencing showed affiliations with 15 previously described and 16 unclassified phyla. The pyrosequencing data were deposited in the NCBI Sequence Read Archive (SRA) under the accession number PRJNA494795. The greatest diversity was observed in the phyla *Acidobacteria* (43.79%) and *Proteobacteria* (29.70%). The other known phyla detected from forest soil were *Chloroflexi*, *Bacteroidetes*, *Actinobacteria*, *Nitrospirae*, *Planctomycetes*, *Gemmatimonadetes*, *Verrucomicrobia*, *Elusimicrobia*, *Chlorobi*, *Cyanobacteria*, *Armatimonadetes*, *Fusobacteria*, and *Fibrobacteres* (Fig. 3). Furthermore, the pyrosequencing data revealed that of the 565 genera in the forest soil sample, 41 had a previously established taxonomic status. The taxonomically known genera with more than 1% diversity included *Koribacter* (8.0%), *Solibacter* (2.0%), *Nitrospira* (2.0%), *Pseudolabrys* (1.0%), *Rhizomicrobium* (1.0%), *Pedosphaera* (1.0%), *Rudaea* (1.0%), *Gaiella* (1.0%), *Afipia* (1.0%), *Telmatobacter* (1.0), and *Geobacter* (1.0%). The culture-independent technique demonstrated the presence of 31 phyla in the analysed soil samples. However, both the novel and traditional cultivation techniques described in this study recovered only four phyla, *Proteobacteria*, *Firmicutes*, *Actinobacteria*, and *Bacteroidetes*. The bacterial diversity of pure cultured isolates was distributed among eight classes, including *γ-Proteobacteria* (21.0%), *Firmicutes* (20.0%), *β*-*Proteobacteria* (19.0%), *α-Proteobacteria* (18.0%), *Actinobacteria* (14.0%), *Sphingobacteria* (14.0%), *Flavobacteria* (3.0%), and *Cytophagia* (1.0%) (Fig. 4).

### Comparison of bacterial diversity recovered by the novel cultivation technique and the traditional method

**Figure 5:**
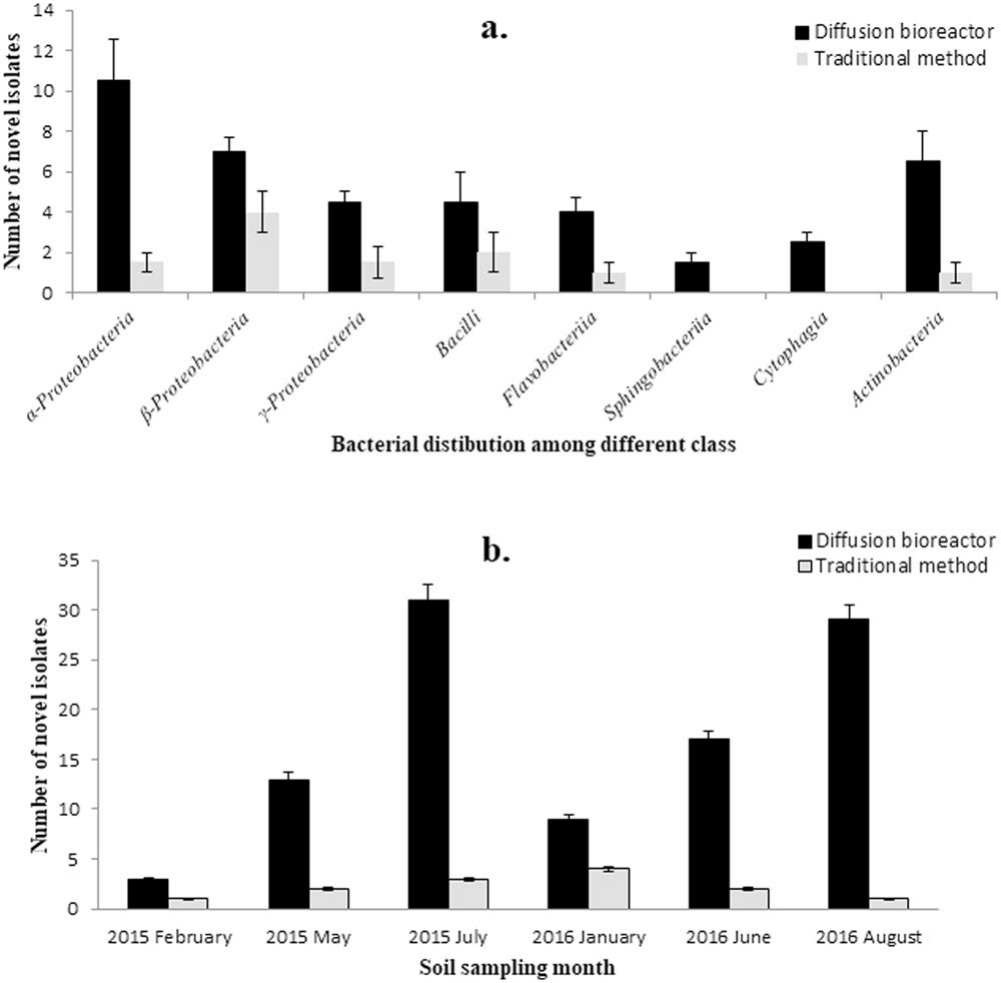
Comparison of the recovery rate of bacterial isolates using the diffusion bioreactor and the traditional method. (**a**) Comparison of previously uncultured and novel isolates obtained using the diffusion bioreactor and traditional method; (**b**) effect of soil sampling period in the recovery of novel/uncultured isolates using both the diffusion bioreactor and the traditional method. The bars represent the standard error of the replicate experiments with duplicate samples.

The newly designed diffusion bioreactor recovered a significantly higher number of novel isolates and previously uncultured bacteria compared to the traditional method [*p* < 0.001; *F*_calculated_ = 19.54 > *F*_critical_; degree of freedom (*df*) = 7]. In the present study, isolates with a 16 S rRNA gene sequence similarity below the threshold value of 98.7–99.0% were considered to be novel strains^18,19^, whereas isolates affiliated with the previously uncultured strains during 16S rRNA gene sequence analysis were considered to be uncultured bacteria. Novel bacterial isolates belonging to the taxa *α-Proteobacteria*, *β*-*Proteobacteria*, *γ-Proteobacteria*, *Firmicutes*, *Flavobacteria*, and *Actinobacteria* were isolated in greater numbers using the diffusion bioreactor technique (67 novel strains and 35 previously uncultured strains were recovered) compared to the traditional method (13 novel strains and no any previously uncultured strains were recovered). The greater number of novel isolates obtained using the new cultivation technique were predominantly from the class *α-Proteobacteria*. Strains belonging to all eight identified classes were isolated by the diffusion bioreactor technique, whereas the use of the traditional method failed to recover representatives of all eight classes. No representative belonging to the classes *Sphingobacteria* and *Cytophagia* were cultured using the traditional method, whereas a large number of bacterial isolates belonging to these classes were successfully cultivated by the newly designed diffusion bioreactor (Fig. 5a).

In this study, the recovery of bacterial diversity across different sampling seasons was evaluated. A noticeable difference was observed in the isolation of bacterial communities in different sampling months. Statistical analysis revealed that the overall recovery of bacterial isolates by both novel and traditional cultivation techniques significantly varied according to the sampling season [*p* < 0.001; *F*_calculated_ = 233.34 > *F*_critical_; degree of freedom (*df*) = 5]. The results that are presented in Fig. 5b show a marked difference in the isolation of bacterial strains between the summer and winter seasons by the diffusion bioreactor. The summer soil samples (May, June, July, and August) yielded a higher number of novel strains compared to the winter samples (January and February). The highest number of novel bacterial isolates (31) was recovered from soil sampled in the month of July by the novel cultivation technique. In contrast, the use of the traditional method resulted in virtually no difference in the isolation of novel strains between the summer and winter soil samples. However, the use of the novel cultivation method resulted in a higher number of novel isolates being obtained from both summer and winter soils compared to the traditional method (Fig. 5b).

**Figure 6:**
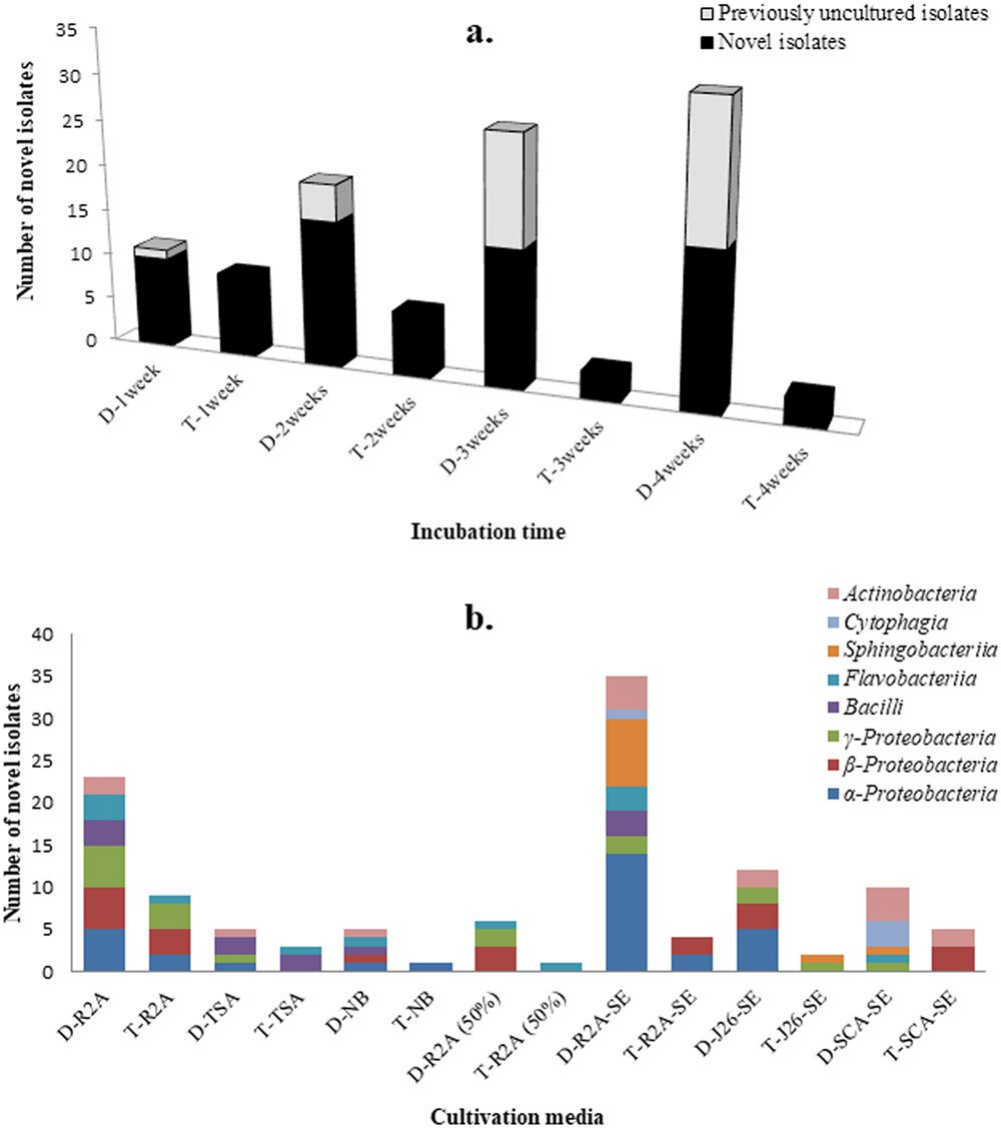
Evaluation of incubation time and medium composition on the recovery of novel/uncultured isolates. (**a**) Effect of incubation period; (**b**) effect of various media used during the cultivation period. D: Represents diffusion bioreactor; and T: represents the traditional method. In this study, isolates sharing a 16S rRNA gene sequence similarity below the threshold value of 98.7–99.0% were considered to be novel strains, and isolates affiliated with the previously uncultured strains during the 16S rRNA gene sequence analysis were considered to be previously uncultured bacteria.

When the incubation time was prolonged, the proportion of previously uncultured strains and novel bacteria isolated using the diffusion bioreactor method was significantly higher than that obtained using the traditional method [*p* < 0.001; *F*_calculated_ = 93.47 > *F*_critical_; degree of freedom (*df*) = 3]. Using the traditional method, a considerable increase in the number of novel isolates obtained was observed during the first week of incubation, whereas no previously uncultured bacteria were cultivated. As the incubation time increased, the yield of novel isolates decreased, and very few novel isolates were cultivated after four weeks by the traditional method. In contrast, the use of the newly developed diffusion bioreactor resulted in the opposite trend, where the yield of both previously uncultured and novel bacterial strains increased as the incubation period increased. The greatest number of previously uncultured bacteria (>30 isolates) and novel microorganisms (>80 isolates) were isolated after four weeks of incubation using the diffusion bioreactor (Fig. 6a).

In this study, the significance of the media composition for isolating novel and uncultured bacteria was evaluated. The results depicted in Fig. 6b shows that nutritionally rich conventional media were significantly poor performers in yielding novel and uncultured bacteria from soil compared to the nutritionally poor modified medium [*p* < 0.001; *F*_calculated_ = 93.88 > *F*_critical_; degree of freedom (*df*) = 6]. Among the eight media tested, LB did not yield any novel and previously uncultured bacteria using either the traditional method or the diffusion bioreactor technique. The modified medium R2A-SE yielded the most diverse range of novel and previously uncultured bacteria from the diffusion bioreactor. All of the assays using modified medium containing soil extract resulted in increased numbers of bacterial taxa being isolated using the newly developed cultivation technique (Fig. 6b).

### Taxonomic analysis of pure culture isolates

**Figure 7:**
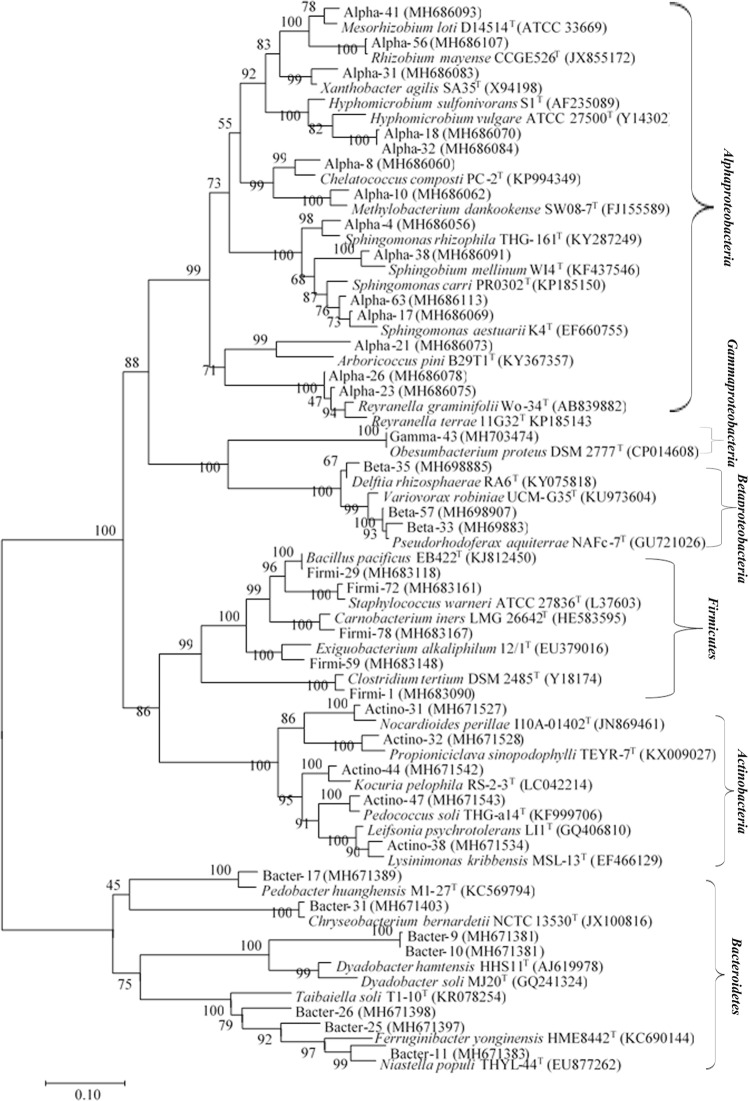
Phylogenetic tree (maximum likelihood tree) based on nearly complete 16S rRNA gene sequences showing the taxonomic affiliations of previously uncultured bacteria isolated using the newly developed diffusion bioreactor with representatives of different established phylogenetic groups. The numbers at the nodes indicate the percentage of 1,000 bootstrap replicates. The bacterial taxa depicted in italics represent reference strains. GenBank accession numbers of the 16S rRNA gene sequences are shown in parentheses. The scale bar represents 0.1 substitutions per nucleotide position.

During cultivation, more than 400 pure bacterial strains were isolated, which belonged to eight classes, 21 orders, and 58 families (Table S2). The results of the 16S rRNA gene sequence analysis identified more than 115 novel bacterial isolates that could be assigned to novel taxa (novel families, genera, and species). Among the 115 novel isolates, 35 strains showed no valid affiliation with previously described species and were considered to be previously uncultivated bacteria (Fig. 7). All of these previously uncultured bacteria were isolated from the newly developed diffusion bioreactors and were slow growing, with colony diameters of less than 0.2 mm. Fourteen previously uncultured isolates and 18 novel isolates showed affiliation with *α-Proteobacteria* (Fig. S2). Three previously uncultivated bacteria and 12 novel strains clustered with *β-Proteobacteria* (Fig. S3). Only one previously uncultured bacterium and eight novel isolates were affiliated with *γ-Proteobacteria* (Fig. S4). Five previously uncultured isolates and eight novel isolates were recovered that belonged to the phylum *Firmicutes* (Fig. S5). *Bacteroidetes* accounted for a total of seven previously uncultured strains and 12 novel isolates (Fig. S6). In the phylum *Actinobacteria*, five previously uncultured isolates and nine novel strains were isolated from the diffusion bioreactor (Fig. S7). Overall, the taxonomic analysis showed that 67 novel isolates were recovered from diffusion bioreactors, whereas the use of traditional method resulted in only 13 novel isolates being obtained (Table S3).

## Discussion

The great diversity of uncultured bacteria discovered using molecular approaches has overwhelmed researchers, motivating them to develop effective strategies for the cultivation of previously uncultured bacteria. One effective strategy being used to cultivate not-yet-cultured bacteria is to simulate their natural environment in the laboratory^5,10,30^. Diffusion chambers have been previously used to mimic the natural environment to culture previously uncultured bacteria^30–32^. A diffusion chamber allows for the passage of necessary elements from the soil (natural environments) into the inner chamber containing medium that has been inoculated with soil inoculum for enrichment. This system also permits interactions among various biotic (microbe-microbe interactions) and abiotic factors (nutrients, oxygen, and growth promoters), supporting co-dependent interactions^10,32^. Therefore, to improve cultivation techniques, in this study, a new cultivation tool was developed based on the diffusion chamber technique. The novel diffusion bioreactor constructed in the present study is useful for isolating novel and previously uncultured bacteria.

With regard to the total size of the bioreactor, the membrane pore size, and the use of liquid enrichment media, the diffusion bioreactor used in the present study is distinct from soil diffusion systems described in previous investigations^9,30–32^. In this study, 2-L plastic containers and polycarbonate membranes with 0.4-µm pore size were used to create the diffusion chambers. The use of large bioreactor with large volume of liquid media enhances the enrichment of soil bacteria. During prolonged incubation, a large volume of nutritionally poor enrichment broth in the diffusion bioreactor helps to compensate for the shortage of nutrients in the bioreactor. The diffusion of necessary elements from the natural soil into the chamber depends on the pore size of the diffusion membrane, and the results of previous studies suggest that a polycarbonate membrane with a 0.1–0.4 µm pore size is suitable for the diffusion of necessary growth elements from the soil environment into the diffusion chamber^33,34^. During the experiment, the diffusion chamber was placed inside the larger outer chamber, and the gap between the walls of these both chambers was filled with the same soil that was used to prepare the soil inoculum. This strategy allows the bacteria in the inner chamber to grow in their natural environment.

The pyrosequencing results showed a broad-range of diverse bacterial phyla (31) present in the forest soil sample. However, both our newly developed technique and the traditional technique recovered isolates belonged to only four phyla (*Proteobacteria*, *Firmicutes*, *Actinobacteria*, and *Bacteroidetes*). Bacterial isolates affiliated with these phyla are commonly reported from soil samples^11,35^. Based on the environmental 16S rRNA gene library, *Acidobacteria* was the most abundant taxon present in the soil sample assayed in this study. However, neither the diffusion bioreactor nor the traditional method produced any colony associated with the phylum *Acidobacteria*, possibly due to the absence of the acidic environment required for the optimum growth of *Acidobacteria*. Most *Acidobacteria* members grow at a pH value of 5.79–5.82, which was not maintained in the media used for this study^36^. In addition, modifying the physiochemical parameters of the culture medium, prolonging the incubation time, and altering the incubation temperature helps to achieve the growth of more diverse bacterial strains^10,37^. These strategies contribute to success in mimicking the natural environment to bridge the gaps between culture-independent and culture-dependent techniques^11,34^.

Taxonomic analysis showed that 67 isolates from the diffusion bioreactor were from novel taxa, and 35 isolates were from previously uncultured taxa. Using the traditional method, only 13 isolates from novel taxa were identified, and no isolates were obtained from previously uncultured taxa. For the comparative study, all of the cultivation conditions and media used were identical for both the newly developed and traditional cultivation techniques. However, a greater number of bacterial isolates were recovered using the diffusion bioreactor system. This result shows that the use of the diffusion bioreactor together with a low-nutrient enrichment medium plays a crucial role during the isolation of novel and previously uncultured species. These results also indicate that the diffusion bioreactor constructed in this study efficiently simulates the natural bacterial growth conditions and enriches bacterial communities effectively. A large number of uncultured bacteria isolated in this study belonged to the phylum *Proteobacteria* and *Bacteroidetes*. The data observed in this study are similar to those obtained using other newly developed techniques for unculturable bacteria that are based on a soil diffusion system^31,32^. The maintenance of a more natural environment within the diffusion bioreactor could be an effective strategy to allow for the cultivation of bacteria that were previously uncultured under normal laboratory conditions^5^.

The ability of the new and the traditional methods to recover the bacterial diversity of soil was evaluated during different sampling seasons. The greatest number of novel isolates were obtained using the novel cultivation technique in July. Overall, there was a marked difference in the yield of novel isolates from soil samples taken during different months. The use of soil obtained in the summer months gave rise to greater recovery of bacteria using the diffusion bioreactor compared to that obtained in the winter months, whereas the use of the traditional method resulted in a consistent level of isolation during both the summer and winter seasons. This result shows that the recovery of previously uncultured bacteria by the newly developed diffusion bioreactor is notably affected by seasonal variation. Because soil bacterial communities are heavily influenced by temperature, moisture, and seasonal changes, the ability to recover soil bacteria as pure isolates is seasonally dependent^38,39^.

There are many slow-growing bacteria that predominate in soil environments. These slow-growers are typically overlooked during cultivation studies because of the lack of the appropriate incubation time, causing them to remain uncultured. One approach to isolate these slow-growing uncultured bacteria is to extend the incubation period^10^, which increases the recovery of the missing strains^12^. The success of this approach is supported by the results of the present study presented, where higher yields of both previously uncultured and novel isolates at extended incubation periods were obtained using the newly developed technique. However, these findings are in contrast with those of a previous study in which no correlation between the isolation of novel strains and incubation time was observed^40^. During a study of microbial diversity, Buerger *et al*. concluded that the recovery of novel species relies on the effort of cultivation rather than the time-frame of incubation period and suggested that bacteria spontaneously awaken from dormancy and start multiplying in random fashion^40^. Spontaneous bacterial awakening from dormancy may have occurred in this study in which unique cultivation methods were implemented to promote the recovery of previously uncultured and novel isolates.

Another approach to address the requirements of uncultured bacteria is to use modified or enriched media for cultivation. In the present study, various media were evaluated for the isolation of novel and uncultured bacteria, with the results showing that the highest recovery of bacterial isolates occurred using the R2A-SE modified medium. The other modified low-substrate media (J26-SE and SCA-SE) were also observed to be promising for the isolation of diverse bacteria from soil. Several bacteria are known to be nutrient-specific and require media with a defined chemical composition for their optimal growth^10^. The traditional media used to study soil microbial diversity results in the recovery of low viable cell counts and frequently fails to isolate members of previously uncultured groups^12^. The use of modified low-substrate media has been used to successfully increase the recovery rate of previously uncultured bacteria. In addition, the use of soil extract together with commercial media has also resulted in the successful recovery of previously uncultured bacteria^5,10,12^.

In summary, the diffusion bioreactor developed in this study based on the soil diffusion system mimics the natural environment and allows for the growth of both previously uncultured and novel isolates. This cultivation strategy, combined with the use of extended incubation periods and modified media, is a promising approach to provide full access for the cultivation of a diverse range of soil bacteria. The newly developed diffusion bioreactor enriches bacterial species diversity and facilitates their cultivation on agar plates. In addition, owing to its larger size, long-term incubations without the need to supplement the chamber with additional substrate are possible using the described diffusion bioreactor. Furthermore, the efficiency of this diffusion bioreactor for the recovery of previously uncultured bacteria can be further evaluated using samples from a variety of geographic settings, such as farmland soil, landfill soil, sediments, marine and fresh water, and Arctic and Antarctic samples.

## Supplementary information

Supplementary tables and figures
