# Supplementary material for: Development of a novel cultivation technique for uncultured soil bacteria
Source: Sci Rep. 2019 Apr 30;9:6666. doi: 10.1038/s41598-019-43182-x (PMC6491550; doi:10.1038/s41598-019-43182-x)
Supplement: Supplementary file 1 — Supplementary tables and figures [file 41598_2019_43182_MOESM1_ESM.pdf]

Supplementary Materials

Scientific Reports

Development of a novel cultivation technique for uncultured soil bacteria

Dhiraj Kumar Chaudhary, Altankhuu Khulan, and Jaisoo Kim*

Ecology Laboratory, Department of Life Science, Kyonggi University, Suwon, South Korea

Running title: Development of a novel cultivation technique

***Correspondence:** Jaisoo Kim, Ecology Laboratory, Dept. of Life Science, College of Natural Sciences and Engineering, Kyonggi University, 154-42 Gwanggyosan-Ro, Youngtong-Gu, Suwon, Gyeonggi-Do 16227, South Korea.

Tel: +82-31-249-9648; Fax: +82-31-253-1165; E-mail: jkimtamu@kgu.ac.kr

Table S1. Composition of media used for the cultivation of bacteria.

Composition	Types of culture media (g/L)							
	Conventional media				Modified media			
	R2A	TSA	LB	NB	50% R2A	R2A-SE	J26 -SE	SCA-SE
Beef extract				3.0				
Casein	0.5	17.0	10.0		0.25	0.5		0.3
Enzymatic digest of soya bean		3.0						
Glucose	0.5	2.5		1.0	0.25	0.5	0.5	
Peptone	0.5			15.0	0.25	0.5		
Sodium pyruvate	0.3				0.15	0.3		
Soluble starch	0.5				0.25	0.5		10.0
Yeast extract	0.5		5.0	3.0	0.25	0.5		
NaCl		5.0	10.0	6.0				
$MgSO_4 \cdot 7H_2O$	0.05				0.02	0.05		0.05
$CaCo_3$								0.02
K_2HPO_4	0.3	2.5			0.15	0.3	0.40	2.0
KNO_3								2.0
$FeCl_2 \cdot 7H_2O$							0.01	0.01
$(NH_2)_2HPO_4$							0.02	
Trace element SL-10*							1 ml	
Selenite tugstate#							1 ml	
Soil extract (SE)						1 L	1 L	1 L
Distilled water	1 L	1 L	1 L	1 L	1 L			
Agar (1.5%)/Gelatine (0.8%)	1.5/0.8	1.5/0.8	1.5/0.8	1.5/0.8	1.5/0.8	1.5/0.8	1.5/0.8	1.5/0.8

*HCl, 10 ml; $CoCl_2 \cdot 6H_2O$, 190 mg; $CuCl_2 \cdot 2H_2O$, 2 mg; $FeCl_2 \cdot 4H_2O$, 1.5 g; $NaBO_3$, 6 mg; $MnCl_2 \cdot 4H_2O$, 100 mg; $Na_2MoO_4 \cdot 2H_2O$, 36 mg; $NiCl_2 \cdot 6H_2O$, 24 mg; $ZnCl_2$, 70 mg; distilled water, 990 ml.

#NaOH, 0.5 g; $Na_2SeO_3 \cdot 5H_2O$, 3 mg; $Na_2WO_4 \cdot 2H_2O$, 4 mg; distilled water, 1000 ml.

Note: Heat to boiling until completely dissolved. Sterilized at 121 °C for 15 minutes; pH= 7.2± 0.2 at 25 °C.

Table S2. Taxonomic distribution of isolated bacteria based on 16S rRNA gene sequence analysis.

Phylum	Class	Order	Family	Genus	Known bacteria	Novel bacteria	Unculturable bacteria
Proteobacteria	***α-Proteobacteria***	*Caulobacterales*	*Caulobacteraceae*	*Brevundimonas*		3	
				Caulobacter	3	3	
				Phenylobacterium	2		
		Rhizobiales	*Bradyrhizobiaceae*	*Bradyrhizobium*	2		
				Tardiphaga	2		
			Brucellaceae	*Ochrobactrum*	2		
			Devosia	*Devosia*		1	
				Paradevosia	1		
				Methyloterrigena	1		
			Hyphomicrobiaceae	*Hyphomicrobium*	1		2
			Methylobacteriaceae	*Bosea*	3		
				Chelatococcus			1
				Methylobacterium	4	1	1
				Microvirga		1	
				Psychroglaciecola			
			Phyllobacteriaceae	*Aminobacter*	1		
				Mesorhizobium		1	1
			Prosthecomicrobium	*Kaistia*	1		
			Rhizobiaceae	*Rhizobium*	1		1
			Xanthobacteraceae	*Ancylobacter*		2	
				Xanthobacter			1
		Rhodobacterales	*Rhodobacteraceae*	*Paracoccus*	2	1	
				Rubellimicrobium			
			Acetobacteraceae	*Roseomonas*	1		
			Alysiosphaera	*Geminicoccus*			1
			Reyranella	*Reyranella*			2
			Rhodospirillaceae	*Azospirillum*	1	1	
				Magnetospirillum	3		
		Sphingomonadales	*Sphingomonadaceae*	*Sphingobium*	1		1
				Sphingomonas	2	4	3
				Sphingopyxis	1	1	
			Erythrobacteraceae	*Altererythrobacter*	1		

	β- Proteobacteria	*Burkholderiales*	*Alcaligenaceae*	*Parapusillimonas*	1	1	
			Burkholderiaceae	*Burkholderia*	13	1	
				Paraburkholderia	6		
			Comamonadaceae	*Acidovorax*		1	
				Comamonas	3		
				Delftia			1
				Pseudacidovorax	1		
				Pseudorhodoferax			1
				Ramlibacter		1	
				Variovorax	1	1	
				Xenophilus	3	1	
				Albidiferax			1
			Oxalobacteraceae	*Massilia*	3	3	
				Herbaspirrillim	1		
				Janthinobacterium	2		
			Ralstonia	*Cupriavidus*	8	1	
			Sphaerotilus	*Pelomonas*	2	1	
		Rhodocyclales	*Rhodocyclales*	*Azospira*		1	
	γ-Proteobacteria	*Alteromonadales*	*Shewanellaceae*	*Shewanella*	1		
		Enterobacteriales	*Enterobacteriaceae*	*Citrobacter*	2		
				Enterobacter	3	3	
				Obesumbacterium			1
				Klebsiella	1		
				Lelliottia	10		
				Serratia	8	1	
				Buttiauxella	1		
		Pseudomonadales	*Moraxellaceae*	*Acinetobacter*	3		
				Enhydrobacter	4		
			Pseudomonadaceae	*Pseudomonas*	21	2	
		Vibrionales	*Vibrionaceae*	*Vibrio*	1		
		Xanthomonadales	*Xanthomonadaceae*	*Dokdonella*	1		
				Dyella	2		
				Lysobacter	1	1	
				Pseudoxanthomonas	4		

				Rhodanobacter		1	
				Stenotrophomonas	9		
Firmicutes	***Bacilli***	*Bacillales*	*Bacillaceae*	*Bacillus*	32	4	1
			Exiguobacteriaceae	*Exiguobacterium*	1		1
			Paenibacillaceae	*Brevibacillus*	6		
				Paenibacillus	3	2	
			Planococcaceae	*Lysinibacillus*	7	1	
				Rummeliibacillus	1		
			Staphylococcaceae	*Staphylococcus*	12		1
		Lactobacillales	*Enterococcaceae*	*Enterococcus*	2		
			Leuconostocaceae	*Leuconostoc*	1		
			Streptococcaceae	*Lactococcus*	2		
			Streptococcaceae	*Streptococcus*		1	
			Carnobacteriaceae	*Carnobacterium*			1
		Clostridiales	*Clostridiaceae*	*Clostridium*			1
Actinobacteria	***Actinobacteria***	*Corynebacteriales*	*Corynebacteriaceae*	*Corynebacterium*	1		
			Mycobacteriaceae	*Mycobacterium*	2		
			Nocardiaceae	*Gordonia*	2		
				Rhodococcus	2		
		Micrococcales	*Brevibacteriaceae*	*Brevibacterium*	3	1	
			Dermacoccaceae	*Barrientosiimonas*	1		
				Kytococcus	1		
				Yimella		1	
			Intrasporangiaceae	*Janibacter*	2		
				Phycicoccus			1
				Terrabacter	1	1	
			Microbacteriaceae	*Agromyces*	4		
				Leifsonia		1	
				Leucobacter	1		
				Lysinimonas			1
				Microbacterium	8	3	
			Micrococcaceae	*Arthrobacter*	1		
				Glutiamicibacter	1		
				Kocuria	2		1
				Micrococcus	7	1	
		Propionibacteriales	*Nocardioidaceae*	*Nocardioides*	2		1
			Propionibacteriaceae	*Aestuariimicrobium*	1		
				Propioniciclava			1
		Streptomycetales	*Streptomycetaceae*	*Streptomyces*		1	

**Bacteroides**	**Flavobacteria**	Flavobacteriales	Flavobacteriaceae	*Chryseobacterium*	10	2	1
				Flavobacterium	1	1	
			Brumimicrobiaceae	*Fluviicola*		1	
	**Cytophagia**	Cytophagales	Dyadobacter	*Dyadobacter*	1	1	
				Ravibacter			2
			Siphonobacter	*Siphonobacter*	1	1	
	**Sphingobacteria**	Sphingobacteriales	Chitinophagaceae	*Chitinophaga*	2	2	
				Pseudobacter			1
				Rurimicrobium			1
				Niabella		2	
				Nemorella			1
				Terrimonas		1	
			Sphingobacteriaceae	*Pedobacter*	1	1	1

Table S3. List of novel isolates obtained using the newly developed and traditional cultivation techniques.

Strains	Closest phylogenetic species	Similarity (%)	GenBank accession no.
**Using the newly developed cultivation technique**			
Alpha-5	*Microvirga pakistanensis* NCCP-1258^{T}	98.1	MH686057
Alpha-9	*Afipia massiliensis* CIP 107022^{T}	96.1	MH686061
Alpha-20	*Hyphomicrobium facile* subsp. ureaphilum ATCC 27492^{T}	98.2	MH686072
Alpha-22	*Devosia epidemidihirudinis* E84^{T}	97.9	MH686074
Alpha-28	*Magnetospirillum moscoviense* BB-1^{T}	96.2	MH686080
Alpha-29	*Azospirillum oryzae* COC8^{T}	98.9	MH686081
Alpha-34	*Sphingosinicella vermicomposti* YC7378^{T}	96.5	MH686086
Alpha-37	*Sphingomonas wittichii* RW1^{T}	99.0	MH686089
Alpha-42	*Sphingomonas ginsengisoli* Gsoil 634^{T}	95.9	MH686094
Alpha-43	*Magnetospirillum moscoviense* BB-1^{T}	96.1	MH686095
Alpha-45	*Sphingopyxis chilensis* S37^{T}	98.9	MH686097
Alpha-47	*Brevundimonas viscosa* CGMCC 1.10683^{T}	98.9	MH686098
Alpha-49	*Azospirillum picis* IMMIB Tar-3^{T}	97.0	MH686100
Alpha-51	*Magnetospirillum gryphiswaldense* MSR-1 v2^{T}	96.1	MH686102
Alpha-52	*Caulobacter daechungensis* H-E3-2^{T}	98.0	MH686103
Alpha-55	*Phenylobacterium aquaticum* W2-3-4^{T}	97.8	MH686106
Alpha-57	*Enterovirga rhinocerotis* YIM 100770^{T}	97.9	MH686108
Alpha-67	*Sphingomonas ginsengisoli* Gsoil 634^{T}	98.5	MH686117
Beta-11	*Paraburkholderia humisilvae* Y-12^{T}	98.3	MH698861
Beta-12	*Paraburkholderia tropica* Ppe8^{T}	98.9	MH698862
Beta-19	*Acidovorax anthurii* CFBP 3232^{T}	98.3	MH698869
Beta-24	*Acidovorax anthurii* CFBP 3232^{T}	98.2	MH698874
Beta-28	*Parapusillimonas granuli* Ch07^{T}	98.1	MH698878
Beta-40	*Massilia chloroacetimidivorans* TA-C7e^{T}	98.6	MH698890
Beta-59	*Cupriavidus yeoncheonensis* DCY86^{T}	99.0	MH698909
Beta-61	*Massilia violacea* CAVIO^{T}	97.9	MH698911
Beta-65	*Massilia violacea* CAVIO^{T}	97.9	MH698915
Beta-66	*Parapusillimonas granuli* Ch07^{T}	97.9	MH698916
Beta-74	*Ramlibacter henchirensis* TMB834^{T}	98.2	MH698924
Beta-26	*Comamonas terrigena* NBRC 13299^{T}	99.0	MH698876
Gamma-11	*Stenotrophomonas nitritireducens* DSM 12575^{T}	98.6	MH703442
Gamma-13	*Moraxella osloensis* CCUG 350^{T}	99.0	MH703444
Gamma-15	*Acinetobacter kookii* ANC 4667^{T}	98.8	MH703446

Gamma-28	*Moraxella osloensis* CCUG 350^T	99.0	MH703444
Gamma-30	*Buttiauxella ferragutiae* ATCC 51602^T	98.4	MH703446
Gamma-41	*Pseudomonas oryzae* KCTC 32247^T	98.4	MH703472
Gamma-42	*Citrobacter farmer* CDC 2991-81^T	98.3	MH703473
Gamma-81	*Pseudomonas japonica* NBRC 103040^T	99.0	MH703512
Bacter-1	*Chryseobacterium scophthalmum* DSM 16779^T	98.4	MH671373
Bacter-2	*Chryseobacterium scophthalmum* DSM 16779^T	98.4	MH671374
Bacter-16	*Niabella pedocola* R384^T	98.9	MH671388
Bacter-21	*Chryseobacterium scophthalmum* DSM 16779^T	98.5	MH671393
Bacter-22	*Flavobacterium mizutaii* NCTC 12149^T	99.0	MH671394
Bacter-24	*Chitinophaga varians* 10-7W-9003^T	98.6	MH671396
Bacter-28	*Pedobacter composti* TR6-06^T	98.4	MH671400
Bacter-29	*Chryseobacterium contaminans* DSM 27621^T	98.5	MH671401
Bacter-32	*Chryseobacterium oncorhynchi* 701B-08^T	97.9	MH671404
Bacter-33	*Chryseobacterium viscerum* 687B-08^T	98.9	MH671405
Bacter-34	*Dyadobacter jiangsuensis* L-1^T	98.9	MH671406
Bacter-35	*Chryseobacterium viscerum* 687B-08^T	98.9	MH671407
Actino-2	*Brevibacterium sanguinis* CF63^T	97.3	MH671499
Actino-5	*Streptomyces stelliscabiei* NRRL B-24447^T	97.9	MH671502
Actino-12	*Microbacterium testaceum* DSM 20166^T	99.0	MH671509
Actino-22	*Microbacterium schleiferi* IFO 15075^T	98.9	MH671519
Actino-26	*Microbacterium testaceum* DSM 20166^T	98.9	MH671523
Actino-34	*Brevibacterium casei* NCDO 2048^T	98.9	MH671530
Actino-37	*Calidifontobacter terrae* R161^T	98.9	MH671533
Actino-41	*Microbacterium arabinogalactanolyticum* IFO 14344^T	98.9	MH671537
Actino-55	*Agromyces humatus* CD5^T	99.0	MH671551
Firmi-27	*Lysinibacillus boronitolerans* T-10a^T	97.4	MH683116
Firmi-33	*Bacillus thermotolerans* SGZ-8^T	97.5	MH683122
Firmi-54	*Brevibacillus agri* NRRL NRS-1219^T	99.0	MH683143
Firmi-56	*Bacillus bataviensis* LMG 21833^T	98.6	MH683145
Firmi-61	*Paenibacillus peoriae* DSM 8320^T	98.9	MH683150
Firmi-67	*Bacillus pseudomycoides* DSM 12442^T	98.8	MH683156
Firmi-75	*Bacillus infantis* NRRL B-14911^T	98.9	MH683164
Firmi-80	*Paenibacillus motobuensis* MC10^T	97.9	MH683169
**Using the traditional cultivation technique**			
Beta-1	*Massilia chloroacetimidivorans* TA-C7e^T	99.0	MH698851
Beta-3	*Paraburkholderia hiiakae* I2^T	98.9	MH698853
Beta-20	*Acidovorax anthurii* CFBP 3232^T	98.3	MH698870

Beta-72	*Paraburkholderia paradisi* WA^{T}	97.8	MH698922
Gamma-1	*Enterobacter roggenkampii* EN-117^{T}	99.0	MH703432
Gamma-8	*Enterobacter roggenkampii* EN-117^{T}	99.0	MH703439
Gamma-22	*Pseudomonas nitroreducens* DSM 14399^{T}	98.9	MH703453
Gamma-33	*Lysobacter dokdonensis* DS-58^{T}	99.0	MH703464
Gamma-34	*Pseudomonas oryzae* KCTC 32247^{T}	98.5	MH703465
Gamma-38	*Pseudomonas oryzae* KCTC 32247^{T}	98.5	MH703469
Bacter-4	*Chitinophaga varians* 10-7W-9003^{T}	98.5	MH671376
Bacter-5	*Chitinophaga varians* 10-7W-9003^{T}	98.4	MH671377
Bacter-30	*Flavobacterium cauense* R2A-7^{T}	98.8	MH671402

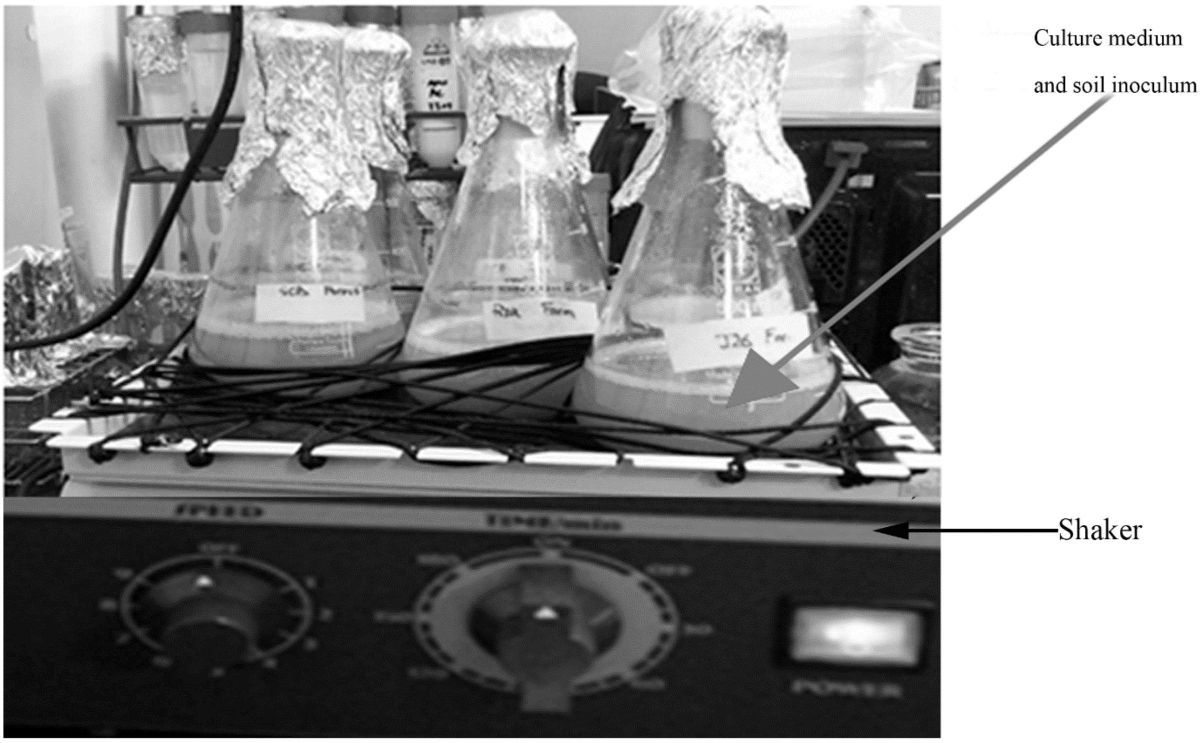

Fig. S1. Experimental setup for the traditional method of isolating soil bacteria. Each conical flask contains the appropriate medium and soil inoculum. All of the flasks were allowed to incubate at room temperature on a shaker for 4 weeks.

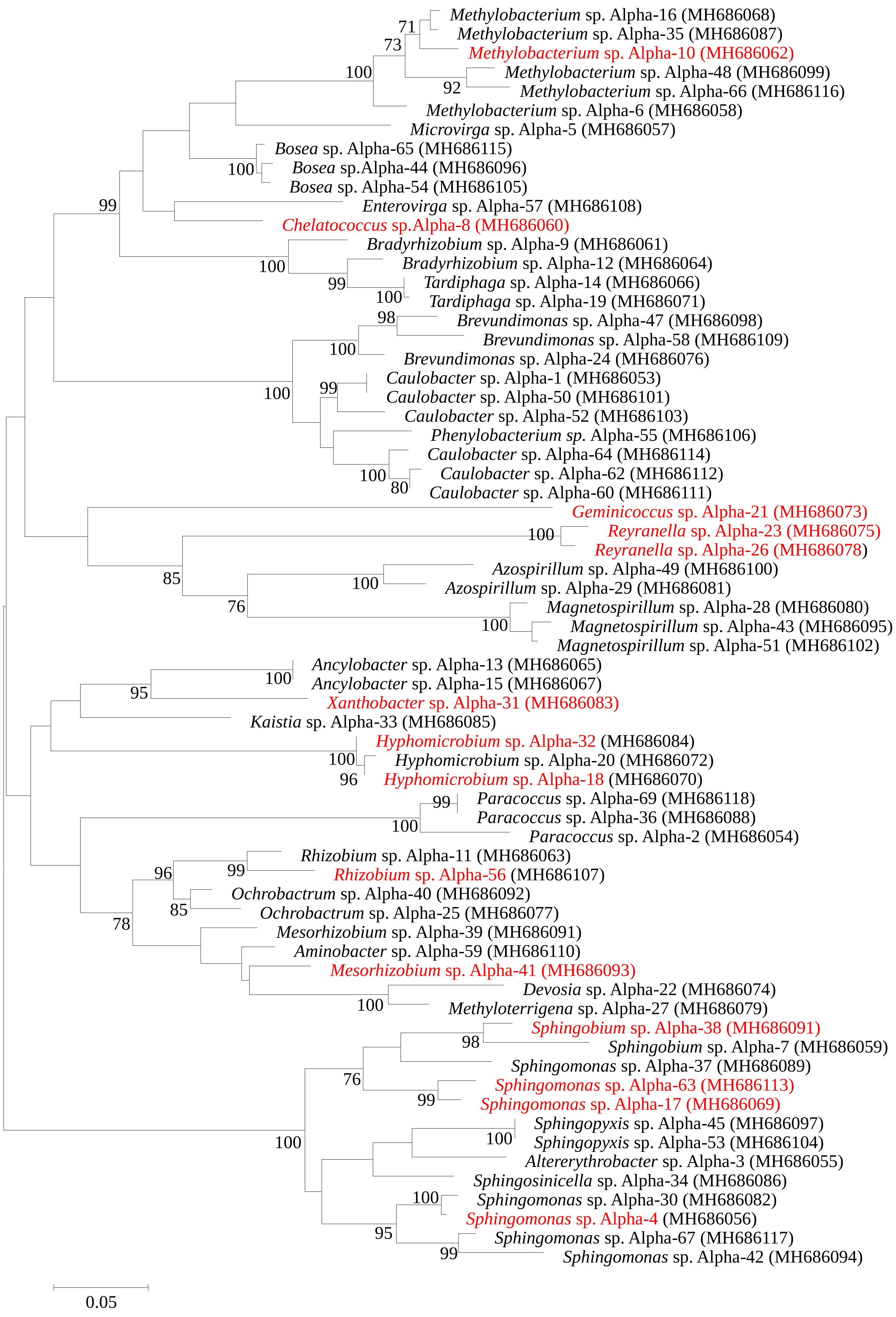

Methylobacterium sp. Alpha-16 (MH686068)
Methylobacterium sp. Alpha-35 (MH686087)
Methylobacterium sp. Alpha-10 (MH686062)
Methylobacterium sp. Alpha-48 (MH686099)
Methylobacterium sp. Alpha-66 (MH686116)
Methylobacterium sp. Alpha-6 (MH686058)
Microvirga sp. Alpha-5 (MH686057)
Bosea sp. Alpha-65 (MH686115)
Bosea sp.Alpha-44 (MH686096)
Bosea sp. Alpha-54 (MH686105)
Enterovirga sp. Alpha-57 (MH686108)
Chelatococcus sp.Alpha-8 (MH686060)
Bradyrhizobium sp. Alpha-9 (MH686061)
Bradyrhizobium sp. Alpha-12 (MH686064)
Tardiphaga sp. Alpha-14 (MH686066)
Tardiphaga sp. Alpha-19 (MH686071)
Brevundimonas sp. Alpha-47 (MH686098)
Brevundimonas sp. Alpha-58 (MH686109)
Brevundimonas sp. Alpha-24 (MH686076)
Caulobacter sp. Alpha-1 (MH686053)
Caulobacter sp. Alpha-50 (MH686101)
Caulobacter sp. Alpha-52 (MH686103)
Phenylobacterium sp. Alpha-55 (MH686106)
Caulobacter sp. Alpha-64 (MH686114)
Caulobacter sp. Alpha-62 (MH686112)
Caulobacter sp. Alpha-60 (MH686111)
Geminicoccus sp. Alpha-21 (MH686073)
Reyranella sp. Alpha-23 (MH686075)
Reyranella sp. Alpha-26 (MH686078)
Azospirillum sp. Alpha-49 (MH686100)
Azospirillum sp. Alpha-29 (MH686081)
Magnetospirillum sp. Alpha-28 (MH686080)
Magnetospirillum sp. Alpha-43 (MH686095)
Magnetospirillum sp. Alpha-51 (MH686102)
Ancylobacter sp. Alpha-13 (MH686065)
Ancylobacter sp. Alpha-15 (MH686067)
Xanthobacter sp. Alpha-31 (MH686083)
Kaistia sp. Alpha-33 (MH686085)
Hyphomicrobium sp. Alpha-32 (MH686084)
Hyphomicrobium sp. Alpha-20 (MH686072)
Hyphomicrobium sp. Alpha-18 (MH686070)
Paracoccus sp. Alpha-69 (MH686118)
Paracoccus sp. Alpha-36 (MH686088)
Paracoccus sp. Alpha-2 (MH686054)
Rhizobium sp. Alpha-11 (MH686063)
Rhizobium sp. Alpha-56 (MH686107)
Ochrobactrum sp. Alpha-40 (MH686092)
Ochrobactrum sp. Alpha-25 (MH686077)
Mesorhizobium sp. Alpha-39 (MH686091)
Aminobacter sp. Alpha-59 (MH686110)
Mesorhizobium sp. Alpha-41 (MH686093)
Devosia sp. Alpha-22 (MH686074)
Methyloterrigena sp. Alpha-27 (MH686079)
Sphingobium sp. Alpha-38 (MH686091)
Sphingobium sp. Alpha-7 (MH686059)
Sphingomonas sp. Alpha-37 (MH686089)
Sphingomonas sp. Alpha-63 (MH686113)
Sphingomonas sp. Alpha-17 (MH686069)
Sphingopyxis sp. Alpha-45 (MH686097)
Sphingopyxis sp. Alpha-53 (MH686104)
Altererythrobacter sp. Alpha-3 (MH686055)
Sphingosinicella sp. Alpha-34 (MH686086)
Sphingomonas sp. Alpha-30 (MH686082)
Sphingomonas sp. Alpha-4 (MH686056)
Sphingomonas sp. Alpha-67 (MH686117)
Sphingomonas sp. Alpha-42 (MH686094)
0.05

Fig. S2. Phylogenetic tree (Maximum likelihood tree) based on nearly complete 16S rRNA gene sequences showing the taxonomic position of pure isolates belonging to *Alphaproteobacteria* class. The isolates indicated in red colour are previously uncultured strains. The numbers at the nodes indicate the percentage of 1,000 bootstrap replicates; only values >70% are shown. GenBank accession numbers of 16S rRNA gene sequences are given in parentheses. The scale bar represents 0.05 substitutions per nucleotide position.

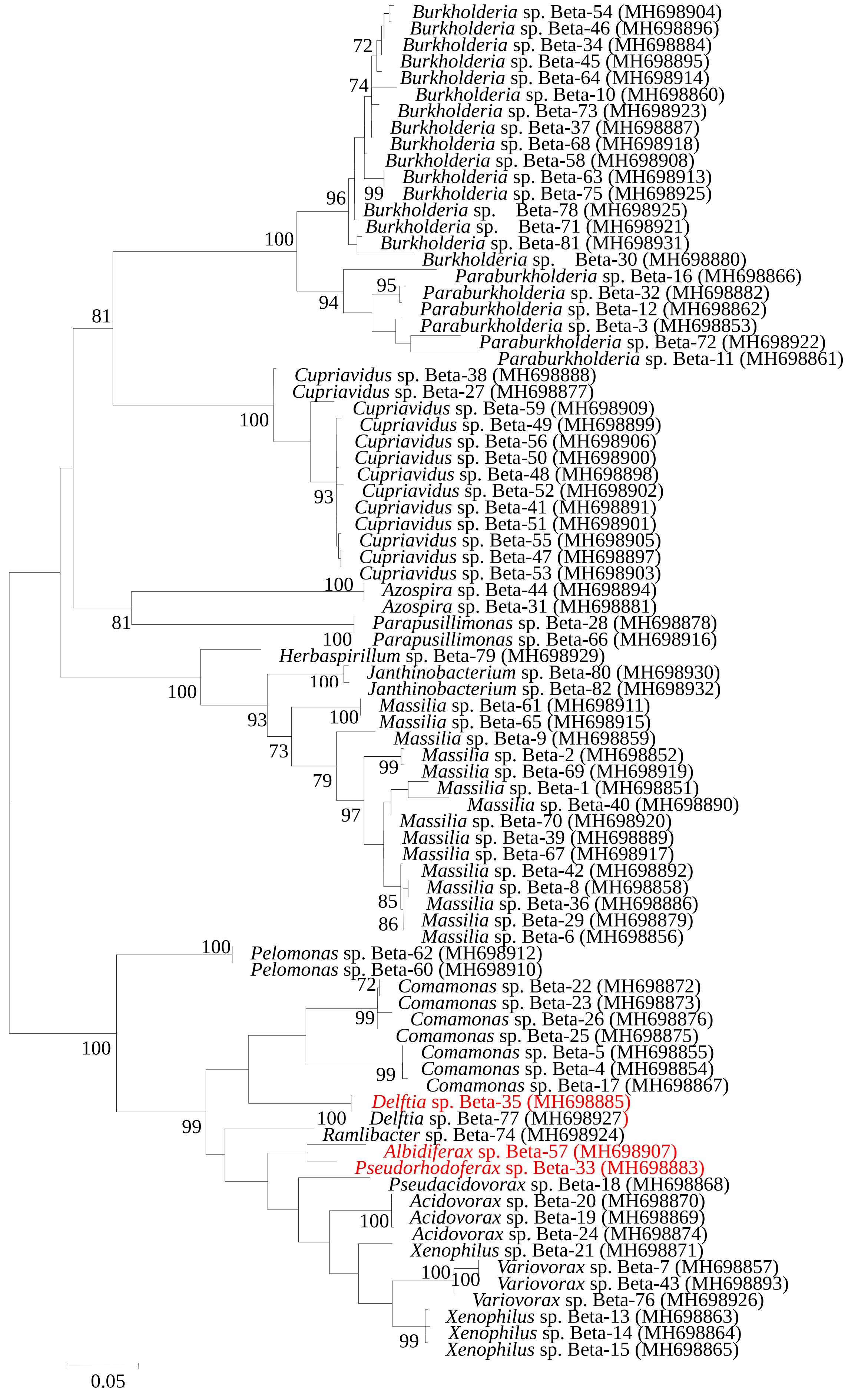

Burkholderia sp. Beta-54 (MH698904)
Burkholderia sp. Beta-46 (MH698896)
Burkholderia sp. Beta-34 (MH698884)
Burkholderia sp. Beta-45 (MH698895)
Burkholderia sp. Beta-64 (MH698914)
Burkholderia sp. Beta-10 (MH698860)
Burkholderia sp. Beta-73 (MH698923)
Burkholderia sp. Beta-37 (MH698887)
Burkholderia sp. Beta-68 (MH698918)
Burkholderia sp. Beta-58 (MH698908)
Burkholderia sp. Beta-63 (MH698913)
Burkholderia sp. Beta-75 (MH698925)
Burkholderia sp. Beta-78 (MH698925)
Burkholderia sp. Beta-71 (MH698921)
Burkholderia sp. Beta-81 (MH698931)
Burkholderia sp. Beta-30 (MH698880)
Paraburkholderia sp. Beta-16 (MH698866)
Paraburkholderia sp. Beta-32 (MH698882)
Paraburkholderia sp. Beta-12 (MH698862)
Paraburkholderia sp. Beta-3 (MH698853)
Paraburkholderia sp. Beta-72 (MH698922)
Paraburkholderia sp. Beta-11 (MH698861)
Cupriavidus sp. Beta-38 (MH698888)
Cupriavidus sp. Beta-27 (MH698877)
Cupriavidus sp. Beta-59 (MH698909)
Cupriavidus sp. Beta-49 (MH698899)
Cupriavidus sp. Beta-56 (MH698906)
Cupriavidus sp. Beta-50 (MH698900)
Cupriavidus sp. Beta-48 (MH698898)
Cupriavidus sp. Beta-52 (MH698902)
Cupriavidus sp. Beta-41 (MH698891)
Cupriavidus sp. Beta-51 (MH698901)
Cupriavidus sp. Beta-55 (MH698905)
Cupriavidus sp. Beta-47 (MH698897)
Cupriavidus sp. Beta-53 (MH698903)
Azospira sp. Beta-44 (MH698894)
Azospira sp. Beta-31 (MH698881)
Parapusillimonas sp. Beta-28 (MH698878)
Parapusillimonas sp. Beta-66 (MH698916)
Herbaspirillum sp. Beta-79 (MH698929)
Janthinobacterium sp. Beta-80 (MH698930)
Janthinobacterium sp. Beta-82 (MH698932)
Massilia sp. Beta-61 (MH698911)
Massilia sp. Beta-65 (MH698915)
Massilia sp. Beta-9 (MH698859)
Massilia sp. Beta-2 (MH698852)
Massilia sp. Beta-69 (MH698919)
Massilia sp. Beta-1 (MH698851)
Massilia sp. Beta-40 (MH698890)
Massilia sp. Beta-70 (MH698920)
Massilia sp. Beta-39 (MH698889)
Massilia sp. Beta-67 (MH698917)
Massilia sp. Beta-42 (MH698892)
Massilia sp. Beta-8 (MH698858)
Massilia sp. Beta-36 (MH698886)
Massilia sp. Beta-29 (MH698879)
Massilia sp. Beta-6 (MH698856)
Pelomonas sp. Beta-62 (MH698912)
Pelomonas sp. Beta-60 (MH698910)
Comamonas sp. Beta-22 (MH698872)
Comamonas sp. Beta-23 (MH698873)
Comamonas sp. Beta-26 (MH698876)
Comamonas sp. Beta-25 (MH698875)
Comamonas sp. Beta-5 (MH698855)
Comamonas sp. Beta-4 (MH698854)
Comamonas sp. Beta-17 (MH698867)
Delftia sp. Beta-35 (MH698885)
Delftia sp. Beta-77 (MH698927)
Ramlibacter sp. Beta-74 (MH698924)
Albidiferax sp. Beta-57 (MH698907)
Pseudorhodoferax sp. Beta-33 (MH698883)
Pseudacidovorax sp. Beta-18 (MH698868)
Acidovorax sp. Beta-20 (MH698870)
Acidovorax sp. Beta-19 (MH698869)
Acidovorax sp. Beta-24 (MH698874)
Xenophilus sp. Beta-21 (MH698871)
Variovorax sp. Beta-7 (MH698857)
Variovorax sp. Beta-43 (MH698893)
Variovorax sp. Beta-76 (MH698926)
Xenophilus sp. Beta-13 (MH698863)
Xenophilus sp. Beta-14 (MH698864)
Xenophilus sp. Beta-15 (MH698865)
0.05

Fig. S3. Phylogenetic tree (Maximum likelihood tree) based on nearly complete 16S rRNA gene sequences showing the taxonomic position of pure isolates belonging to *Betaproteobacteria* class. The isolates indicated in red colour are previously uncultured strains. The numbers at the nodes indicate the percentage of 1,000 bootstrap replicates; only values >70% are shown. GenBank accession numbers of 16S rRNA gene sequences are given in parentheses. The scale bar represents 0.05 substitutions per nucleotide position.

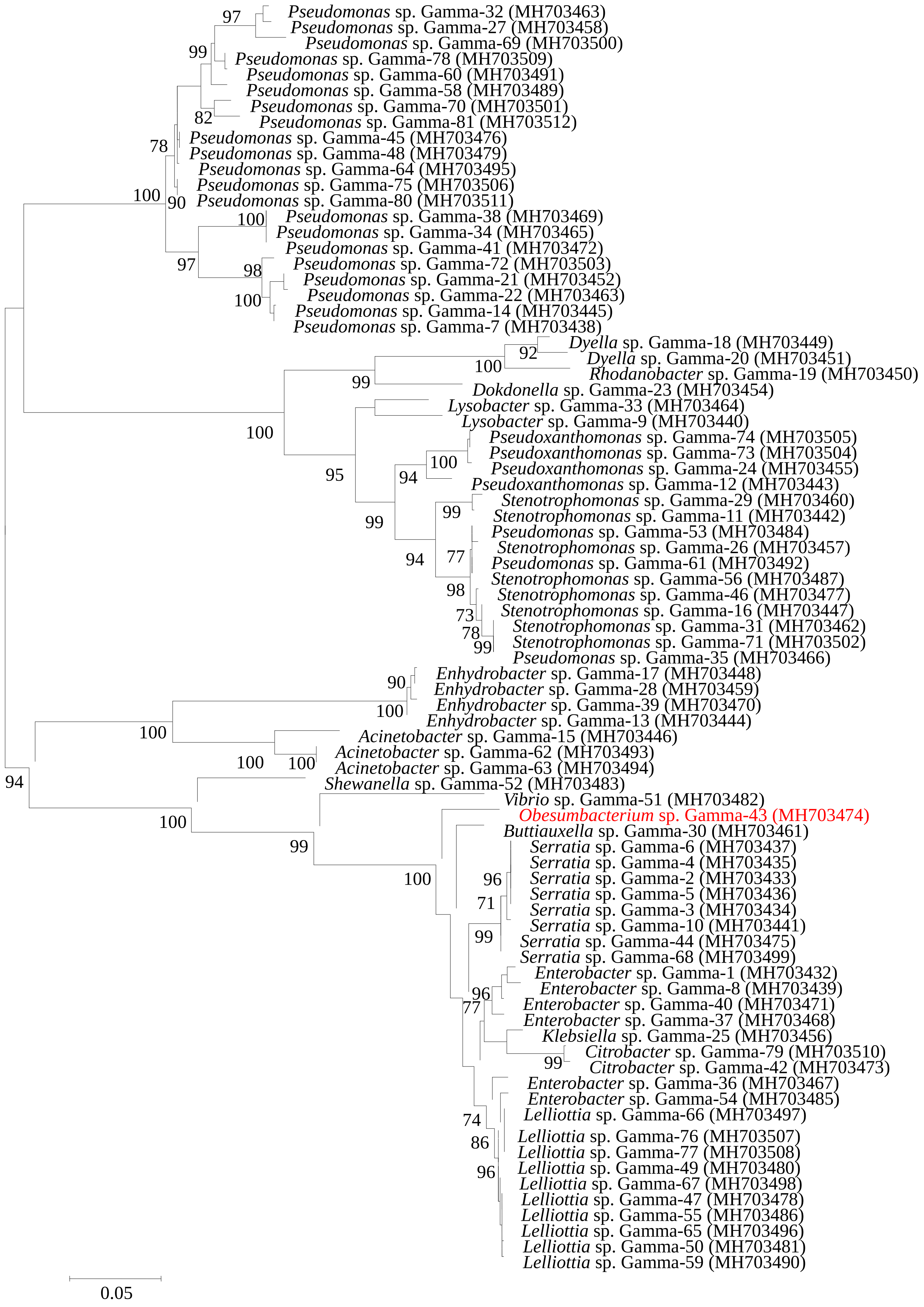

Pseudomonas sp. Gamma-32 (MH703463)
Pseudomonas sp. Gamma-27 (MH703458)
Pseudomonas sp. Gamma-69 (MH703500)
Pseudomonas sp. Gamma-78 (MH703509)
Pseudomonas sp. Gamma-60 (MH703491)
Pseudomonas sp. Gamma-58 (MH703489)
Pseudomonas sp. Gamma-70 (MH703501)
Pseudomonas sp. Gamma-81 (MH703512)
Pseudomonas sp. Gamma-45 (MH703476)
Pseudomonas sp. Gamma-48 (MH703479)
Pseudomonas sp. Gamma-64 (MH703495)
Pseudomonas sp. Gamma-75 (MH703506)
Pseudomonas sp. Gamma-80 (MH703511)
Pseudomonas sp. Gamma-38 (MH703469)
Pseudomonas sp. Gamma-34 (MH703465)
Pseudomonas sp. Gamma-41 (MH703472)
Pseudomonas sp. Gamma-72 (MH703503)
Pseudomonas sp. Gamma-21 (MH703452)
Pseudomonas sp. Gamma-22 (MH703463)
Pseudomonas sp. Gamma-14 (MH703445)
Pseudomonas sp. Gamma-7 (MH703438)
Dyella sp. Gamma-18 (MH703449)
Dyella sp. Gamma-20 (MH703451)
Rhodanobacter sp. Gamma-19 (MH703450)
Dokdonella sp. Gamma-23 (MH703454)
Lysobacter sp. Gamma-33 (MH703464)
Lysobacter sp. Gamma-9 (MH703440)
Pseudoxanthomonas sp. Gamma-74 (MH703505)
Pseudoxanthomonas sp. Gamma-73 (MH703504)
Pseudoxanthomonas sp. Gamma-24 (MH703455)
Pseudoxanthomonas sp. Gamma-12 (MH703443)
Stenotrophomonas sp. Gamma-29 (MH703460)
Stenotrophomonas sp. Gamma-11 (MH703442)
Pseudomonas sp. Gamma-53 (MH703484)
Stenotrophomonas sp. Gamma-26 (MH703457)
Pseudomonas sp. Gamma-61 (MH703492)
Stenotrophomonas sp. Gamma-56 (MH703487)
Stenotrophomonas sp. Gamma-46 (MH703477)
Stenotrophomonas sp. Gamma-16 (MH703447)
Stenotrophomonas sp. Gamma-31 (MH703462)
Stenotrophomonas sp. Gamma-71 (MH703502)
Pseudomonas sp. Gamma-35 (MH703466)
Enhydrobacter sp. Gamma-17 (MH703448)
Enhydrobacter sp. Gamma-28 (MH703459)
Enhydrobacter sp. Gamma-39 (MH703470)
Enhydrobacter sp. Gamma-13 (MH703444)
Acinetobacter sp. Gamma-15 (MH703446)
Acinetobacter sp. Gamma-62 (MH703493)
Acinetobacter sp. Gamma-63 (MH703494)
Shewanella sp. Gamma-52 (MH703483)
Vibrio sp. Gamma-51 (MH703482)
Obesumbacterium sp. Gamma-43 (MH703474)
Buttiauxella sp. Gamma-30 (MH703461)
Serratia sp. Gamma-6 (MH703437)
Serratia sp. Gamma-4 (MH703435)
Serratia sp. Gamma-2 (MH703433)
Serratia sp. Gamma-5 (MH703436)
Serratia sp. Gamma-3 (MH703434)
Serratia sp. Gamma-10 (MH703441)
Serratia sp. Gamma-44 (MH703475)
Serratia sp. Gamma-68 (MH703499)
Enterobacter sp. Gamma-1 (MH703432)
Enterobacter sp. Gamma-8 (MH703439)
Enterobacter sp. Gamma-40 (MH703471)
Enterobacter sp. Gamma-37 (MH703468)
Klebsiella sp. Gamma-25 (MH703456)
Citrobacter sp. Gamma-79 (MH703510)
Citrobacter sp. Gamma-42 (MH703473)
Enterobacter sp. Gamma-36 (MH703467)
Enterobacter sp. Gamma-54 (MH703485)
Lelliottia sp. Gamma-66 (MH703497)
Lelliottia sp. Gamma-76 (MH703507)
Lelliottia sp. Gamma-77 (MH703508)
Lelliottia sp. Gamma-49 (MH703480)
Lelliottia sp. Gamma-67 (MH703498)
Lelliottia sp. Gamma-47 (MH703478)
Lelliottia sp. Gamma-55 (MH703486)
Lelliottia sp. Gamma-65 (MH703496)
Lelliottia sp. Gamma-50 (MH703481)
Lelliottia sp. Gamma-59 (MH703490)
0.05

Fig. S4. Phylogenetic tree (Maximum likelihood tree) based on nearly complete 16S rRNA gene sequences showing the taxonomic position of pure isolates belonging to *Gammaproteobacteria* class. The isolate indicated in red colour is previously uncultured strain. The numbers at the nodes indicate the percentage of 1,000 bootstrap replicates; only values >70% are shown. GenBank accession numbers of 16S rRNA gene sequences are given in parentheses. The scale bar represents 0.05 substitutions per nucleotide position.

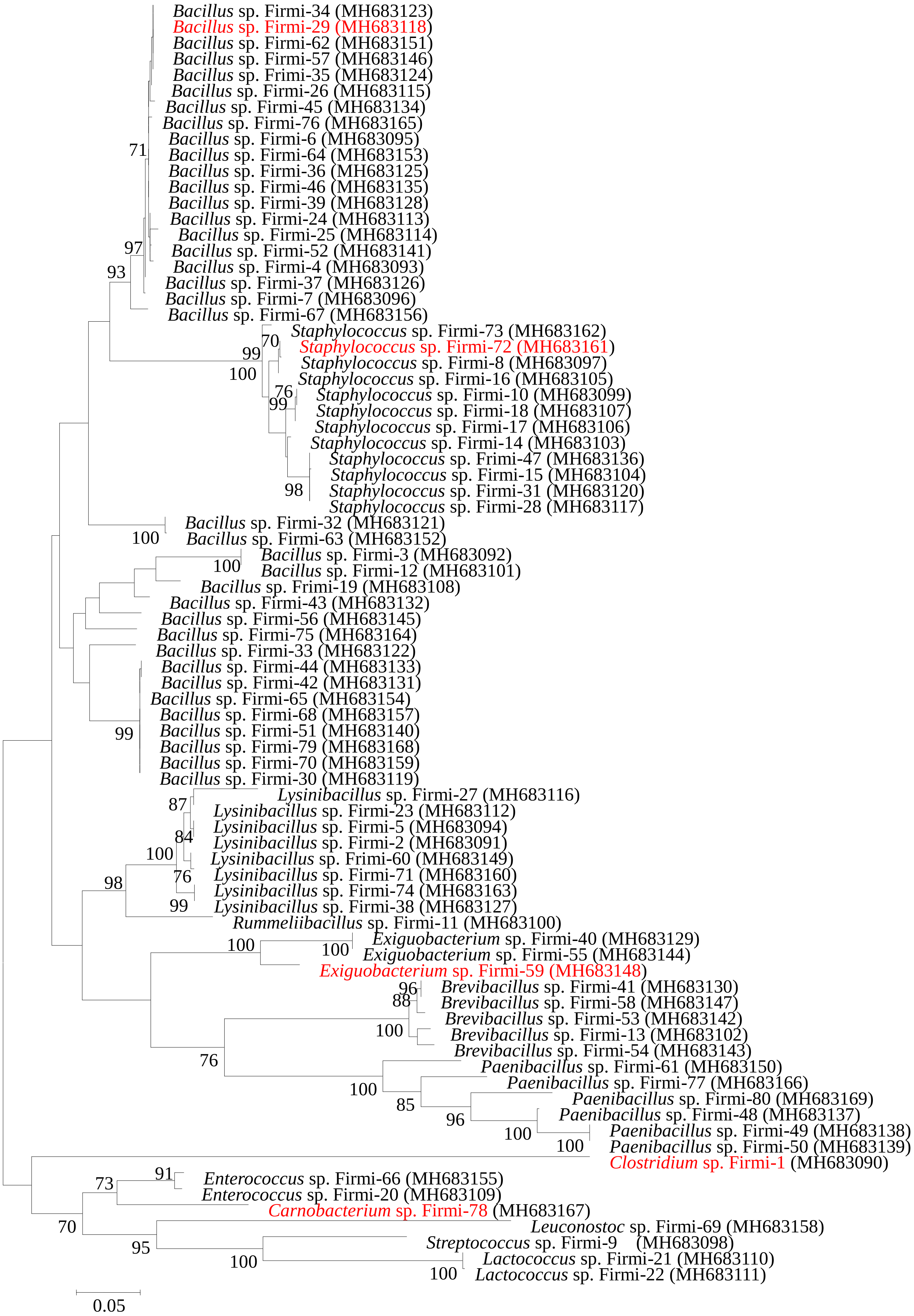
Bacillus sp. Firmi-34 (MH683123)
Bacillus sp. Firmi-29 (MH683118)
Bacillus sp. Firmi-62 (MH683151)
Bacillus sp. Firmi-57 (MH683146)
Bacillus sp. Frimi-35 (MH683124)
Bacillus sp. Firmi-26 (MH683115)
Bacillus sp. Firmi-45 (MH683134)
Bacillus sp. Firmi-76 (MH683165)
Bacillus sp. Firmi-6 (MH683095)
Bacillus sp. Firmi-64 (MH683153)
Bacillus sp. Firmi-36 (MH683125)
Bacillus sp. Firmi-46 (MH683135)
Bacillus sp. Firmi-39 (MH683128)
Bacillus sp. Firmi-24 (MH683113)
Bacillus sp. Firmi-25 (MH683114)
Bacillus sp. Firmi-52 (MH683141)
Bacillus sp. Firmi-4 (MH683093)
Bacillus sp. Firmi-37 (MH683126)
Bacillus sp. Firmi-7 (MH683096)
Bacillus sp. Firmi-67 (MH683156)
Staphylococcus sp. Firmi-73 (MH683162)
Staphylococcus sp. Firmi-72 (MH683161)
Staphylococcus sp. Firmi-8 (MH683097)
Staphylococcus sp. Firmi-16 (MH683105)
Staphylococcus sp. Firmi-10 (MH683099)
Staphylococcus sp. Firmi-18 (MH683107)
Staphylococcus sp. Firmi-17 (MH683106)
Staphylococcus sp. Firmi-14 (MH683103)
Staphylococcus sp. Frimi-47 (MH683136)
Staphylococcus sp. Firmi-15 (MH683104)
Staphylococcus sp. Firmi-31 (MH683120)
Staphylococcus sp. Firmi-28 (MH683117)
Bacillus sp. Firmi-32 (MH683121)
Bacillus sp. Firmi-63 (MH683152)
Bacillus sp. Firmi-3 (MH683092)
Bacillus sp. Firmi-12 (MH683101)
Bacillus sp. Frimi-19 (MH683108)
Bacillus sp. Firmi-43 (MH683132)
Bacillus sp. Firmi-56 (MH683145)
Bacillus sp. Firmi-75 (MH683164)
Bacillus sp. Firmi-33 (MH683122)
Bacillus sp. Firmi-44 (MH683133)
Bacillus sp. Firmi-42 (MH683131)
Bacillus sp. Firmi-65 (MH683154)
Bacillus sp. Firmi-68 (MH683157)
Bacillus sp. Firmi-51 (MH683140)
Bacillus sp. Firmi-79 (MH683168)
Bacillus sp. Firmi-70 (MH683159)
Bacillus sp. Firmi-30 (MH683119)
Lysinibacillus sp. Firmi-27 (MH683116)
Lysinibacillus sp. Firmi-23 (MH683112)
Lysinibacillus sp. Firmi-5 (MH683094)
Lysinibacillus sp. Firmi-2 (MH683091)
Lysinibacillus sp. Frimi-60 (MH683149)
Lysinibacillus sp. Firmi-71 (MH683160)
Lysinibacillus sp. Firmi-74 (MH683163)
Lysinibacillus sp. Firmi-38 (MH683127)
Rummeliibacillus sp. Firmi-11 (MH683100)
Exiguobacterium sp. Firmi-40 (MH683129)
Exiguobacterium sp. Firmi-55 (MH683144)
Exiguobacterium sp. Firmi-59 (MH683148)
Brevibacillus sp. Firmi-41 (MH683130)
Brevibacillus sp. Firmi-58 (MH683147)
Brevibacillus sp. Firmi-53 (MH683142)
Brevibacillus sp. Firmi-13 (MH683102)
Brevibacillus sp. Firmi-54 (MH683143)
Paenibacillus sp. Firmi-61 (MH683150)
Paenibacillus sp. Firmi-77 (MH683166)
Paenibacillus sp. Firmi-80 (MH683169)
Paenibacillus sp. Firmi-48 (MH683137)
Paenibacillus sp. Firmi-49 (MH683138)
Paenibacillus sp. Firmi-50 (MH683139)
Clostridium sp. Firmi-1 (MH683090)
Enterococcus sp. Firmi-66 (MH683155)
Enterococcus sp. Firmi-20 (MH683109)
Carnobacterium sp. Firmi-78 (MH683167)
Leuconostoc sp. Firmi-69 (MH683158)
Streptococcus sp. Firmi-9 (MH683098)
Lactococcus sp. Firmi-21 (MH683110)
Lactococcus sp. Firmi-22 (MH683111)
0.05

Fig. S5. Phylogenetic tree (Maximum likelihood tree) based on nearly complete 16S rRNA gene sequences showing the taxonomic position of pure isolates belonging to phylum *Firmicutes*. The isolates indicated in red colour are previously uncultured strains. The numbers at the nodes indicate the percentage of 1,000 bootstrap replicates; only values >70% are shown. GenBank accession numbers of 16S rRNA gene sequences are given in parentheses. The scale bar represents 0.05 substitutions per nucleotide position.

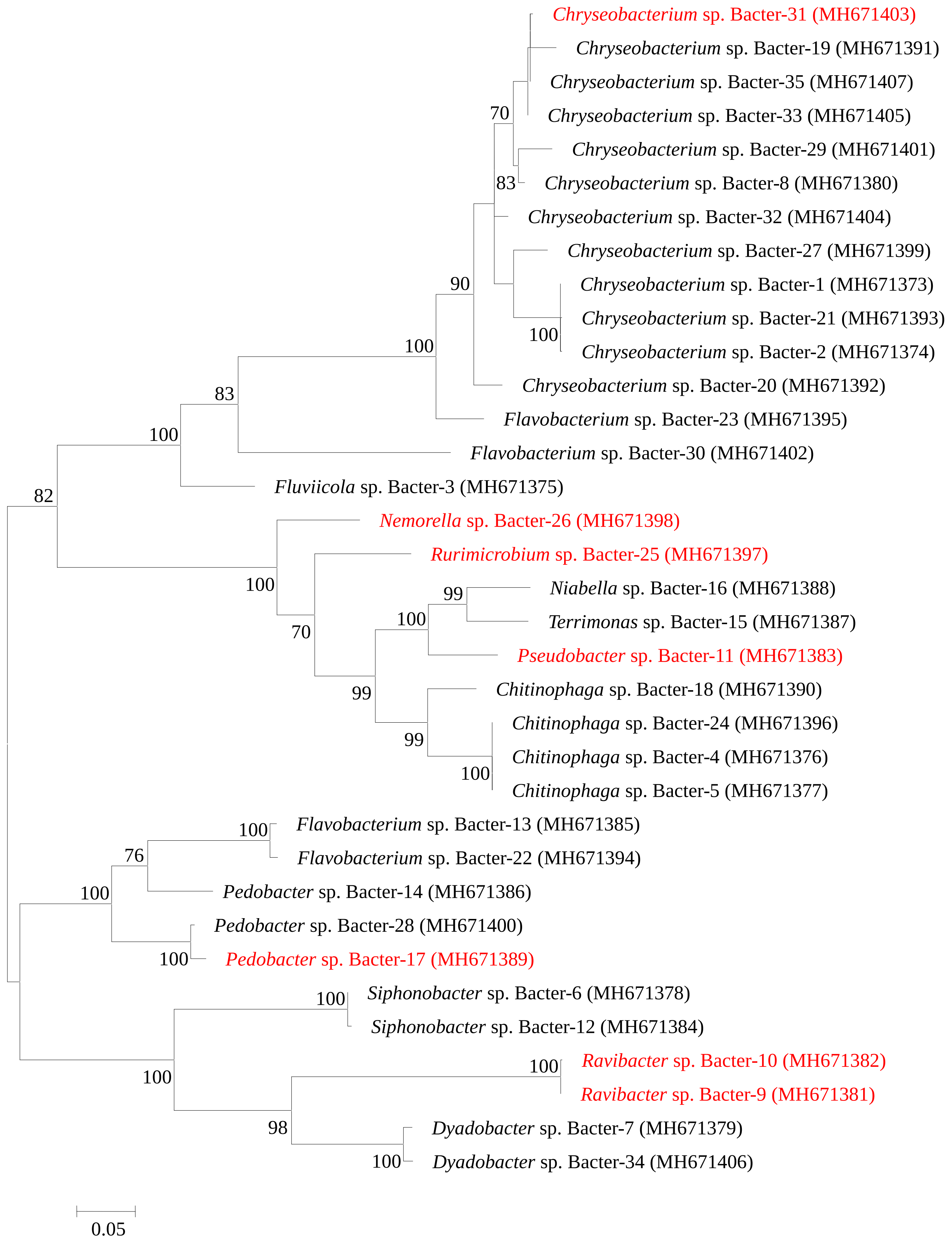
Chryseobacterium sp. Bacter-31 (MH671403)
Chryseobacterium sp. Bacter-19 (MH671391)
Chryseobacterium sp. Bacter-35 (MH671407)
Chryseobacterium sp. Bacter-33 (MH671405)
Chryseobacterium sp. Bacter-29 (MH671401)
Chryseobacterium sp. Bacter-8 (MH671380)
Chryseobacterium sp. Bacter-32 (MH671404)
Chryseobacterium sp. Bacter-27 (MH671399)
Chryseobacterium sp. Bacter-1 (MH671373)
Chryseobacterium sp. Bacter-21 (MH671393)
Chryseobacterium sp. Bacter-2 (MH671374)
Chryseobacterium sp. Bacter-20 (MH671392)
Flavobacterium sp. Bacter-23 (MH671395)
Flavobacterium sp. Bacter-30 (MH671402)
Fluviicola sp. Bacter-3 (MH671375)
Nemorella sp. Bacter-26 (MH671398)
Rurimicrobium sp. Bacter-25 (MH671397)
Niabella sp. Bacter-16 (MH671388)
Terrimonas sp. Bacter-15 (MH671387)
Pseudobacter sp. Bacter-11 (MH671383)
Chitinophaga sp. Bacter-18 (MH671390)
Chitinophaga sp. Bacter-24 (MH671396)
Chitinophaga sp. Bacter-4 (MH671376)
Chitinophaga sp. Bacter-5 (MH671377)
Flavobacterium sp. Bacter-13 (MH671385)
Flavobacterium sp. Bacter-22 (MH671394)
Pedobacter sp. Bacter-14 (MH671386)
Pedobacter sp. Bacter-28 (MH671400)
Pedobacter sp. Bacter-17 (MH671389)
Siphonobacter sp. Bacter-6 (MH671378)
Siphonobacter sp. Bacter-12 (MH671384)
Ravibacter sp. Bacter-10 (MH671382)
Ravibacter sp. Bacter-9 (MH671381)
Dyadobacter sp. Bacter-7 (MH671379)
Dyadobacter sp. Bacter-34 (MH671406)
0.05

Fig. S6. Phylogenetic tree (Maximum likelihood tree) based on nearly complete 16S rRNA gene sequences showing the taxonomic position of pure isolates belonging to phylum *Bacteroidetes*. The isolates indicated in red colour are previously uncultured strains. The numbers at the nodes indicate the percentage of 1,000 bootstrap replicates; only values >70% are shown. GenBank accession numbers of 16S rRNA gene sequences are given in parentheses. The scale bar represents 0.05 substitutions per nucleotide position.

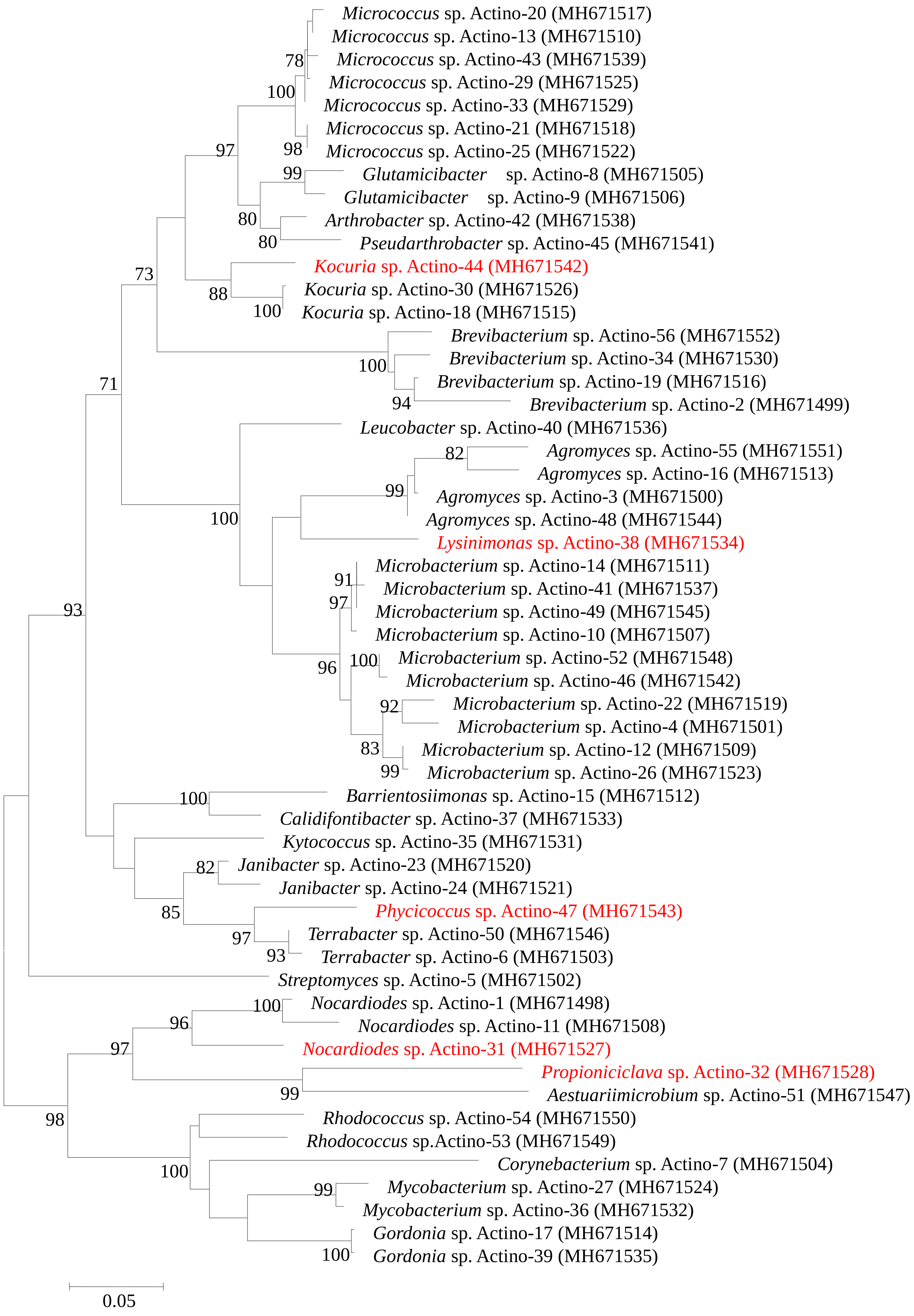
Micrococcus sp. Actino-20 (MH671517)
Micrococcus sp. Actino-13 (MH671510)
Micrococcus sp. Actino-43 (MH671539)
Micrococcus sp. Actino-29 (MH671525)
Micrococcus sp. Actino-33 (MH671529)
Micrococcus sp. Actino-21 (MH671518)
Micrococcus sp. Actino-25 (MH671522)
Glutamicibacter sp. Actino-8 (MH671505)
Glutamicibacter sp. Actino-9 (MH671506)
Arthrobacter sp. Actino-42 (MH671538)
Pseudarthrobacter sp. Actino-45 (MH671541)
Kocuria sp. Actino-44 (MH671542)
Kocuria sp. Actino-30 (MH671526)
Kocuria sp. Actino-18 (MH671515)
Brevibacterium sp. Actino-56 (MH671552)
Brevibacterium sp. Actino-34 (MH671530)
Brevibacterium sp. Actino-19 (MH671516)
Brevibacterium sp. Actino-2 (MH671499)
Leucobacter sp. Actino-40 (MH671536)
Agromyces sp. Actino-55 (MH671551)
Agromyces sp. Actino-16 (MH671513)
Agromyces sp. Actino-3 (MH671500)
Agromyces sp. Actino-48 (MH671544)
Lysinimonas sp. Actino-38 (MH671534)
Microbacterium sp. Actino-14 (MH671511)
Microbacterium sp. Actino-41 (MH671537)
Microbacterium sp. Actino-49 (MH671545)
Microbacterium sp. Actino-10 (MH671507)
Microbacterium sp. Actino-52 (MH671548)
Microbacterium sp. Actino-46 (MH671542)
Microbacterium sp. Actino-22 (MH671519)
Microbacterium sp. Actino-4 (MH671501)
Microbacterium sp. Actino-12 (MH671509)
Microbacterium sp. Actino-26 (MH671523)
Barrientosiimonas sp. Actino-15 (MH671512)
Calidifontibacter sp. Actino-37 (MH671533)
Kytococcus sp. Actino-35 (MH671531)
Janibacter sp. Actino-23 (MH671520)
Janibacter sp. Actino-24 (MH671521)
Phycicoccus sp. Actino-47 (MH671543)
Terrabacter sp. Actino-50 (MH671546)
Terrabacter sp. Actino-6 (MH671503)
Streptomyces sp. Actino-5 (MH671502)
Nocardiodes sp. Actino-1 (MH671498)
Nocardiodes sp. Actino-11 (MH671508)
Nocardiodes sp. Actino-31 (MH671527)
Propioniciclava sp. Actino-32 (MH671528)
Aestuariimicrobium sp. Actino-51 (MH671547)
Rhodococcus sp. Actino-54 (MH671550)
Rhodococcus sp.Actino-53 (MH671549)
Corynebacterium sp. Actino-7 (MH671504)
Mycobacterium sp. Actino-27 (MH671524)
Mycobacterium sp. Actino-36 (MH671532)
Gordonia sp. Actino-17 (MH671514)
Gordonia sp. Actino-39 (MH671535)
0.05

Fig. S7. Phylogenetic tree (Maximum likelihood tree) based on nearly complete 16S rRNA gene sequences showing the taxonomic position of pure isolates belonging to phylum *Actinobacteria*. The isolates indicated in red colour are previously uncultured strains. The numbers at the nodes indicate the percentage of 1,000 bootstrap replicates; only values >70% are shown. GenBank accession numbers of 16S rRNA gene sequences are given in parentheses. The scale bar represents 0.05 substitutions per nucleotide position.
